# Adjuvanted Influenza Vaccines Versus Other Influenza Vaccines in Adults Aged ≥60 Years: A Systematic Review and Meta-Analysis of Relative Effectiveness for Preventing Influenza-Related Hospitalization

**DOI:** 10.3390/vaccines14100883

**Published:** 2026-10-06

**Authors:** Daniela Amicizia, Francesca Marchini, Irene Schenone, Elvira Massaro, Paolo Landa, Aurelien Nicosia, Andrea Orsi, Giancarlo Icardi, Filippo Ansaldi

**Affiliations:** 1 Department of Health Sciences, University of Genoa, 16132 Genoa, Italy; elvira.massaro@unige.it (E.M.); andrea.orsi@unige.it (A.O.); filippo.ansaldi@unige.it (F.A.); 2 IRCCS Azienda Ospedaliera Metropolitana (IRCCS AOM), Ospedale Policlinico San Martino, 16132 Genoa, Italy; francesca.marchini@aomliguria.it (F.M.); irene.schenone@aomliguria.it (I.S.); 3 Département des Opérations et Systèmes de Décision, Faculté des Sciences de L’Administration, Université Laval, Quebec, QC G1V 0A6, Canada; paolo.landa@fsa.ulaval.ca (P.L.); aurelien.nicosia@mat.ulaval.ca (A.N.)

**Keywords:** influenza vaccines, adjuvanted vaccines, non-adjuvanted vaccines, effectiveness

## Abstract

Background/Objectives: Seasonal influenza vaccines have been developed to reduce the risk of severe complications in older adults, who are at increased risk of influenza-related morbidity and mortality. This study aimed to synthesize the available evidence on the comparative effectiveness of MF59-adjuvanted versus high-dose and non-adjuvanted influenza vaccines in preventing hospitalization/ED access. Methods: A systematic literature review of observational and pragmatic studies was conducted in accordance with the PRISMA (Preferred Reporting Items for Systematic Reviews and Meta-Analyses) guidelines. Eligibility was set at ≥60 years. Results: Initially, 1556 records were identified for screening by title and abstract. After removing duplicates (n = 378), 1122 were judged ineligible; 56 records were screened for full-text evaluation. The reconciled dataset contained 13 publications for ADJ versus HD (12 with overall-population estimates and one with risk-stratum estimates) and eight for ADJ versus SD; two publications contributed to both comparisons. All included studies concerned adults aged ≥65 years. In the exploratory overall displays, including explicitly identified broader outcomes, exploratory summaries across adjusted ratio measures were 0.977 (95% normal-theory CI 0.946 to 1.010) for MF59-adjuvanted versus high-dose vaccines (12 publications, 12 estimates; I^2^ = 75.8%; 95% prediction interval 0.891 to 1.072) and 0.818 (95% normal-theory CI 0.783 to 0.855) versus standard-dose vaccines (eight publications, eight estimates; I^2^ = 41.6%; 95% prediction interval 0.758 to 0.883; Hartung–Knapp CI 0.766 to 0.875). Conclusions: The exploratory synthesis did not show a clear difference between MF59-adjuvanted and high-dose vaccines. The average favored adjuvanted over standard-dose vaccines, but sensitivity to outcome selection, heterogeneity estimation and potentially overlapping populations limits interpretation.

## 1. Introduction

Seasonal influenza is a critical public health challenge, causing approximately one billion infections and up to 650,000 respiratory deaths globally each year; in Europe alone, this translates to millions of symptomatic cases and tens of thousands of deaths [1].

While most symptomatic cases of influenza diseases are mild, severe disease is unevenly distributed across the population. Although attack rates are typically highest in children, the clinical and economic burden falls disproportionately on older adults [2]. Individuals aged ≥65 years account for up to 50% of all influenza-related hospitalizations and 90% of associated deaths [3,4]. This heightened vulnerability is driven by immunosenescence—which blunts the immune response to both natural infection and standard vaccination—as well as a higher prevalence of chronic comorbidities [5,6]. Notably, influenza not only causes acute respiratory complications but can also exacerbate pre-existing conditions, leading to permanent functional decline and a loss of independence [7].

As life expectancy rises globally, effective preventive interventions like vaccination against different influenza viruses are essential to reduce influenza-related complications, including hospitalization and death, predominantly among high-risk groups such as older adults, and promote healthy aging [8]. To address the limitations of standard-dose seasonal influenza vaccines (SIVs) in overcoming immunosenescence, the market introduced highly differentiated “enhanced” formulations specifically designed for the elderly [9,10]. These primarily include MF59-adjuvanted vaccines (aTIV, aQIV) and non-adjuvanted high-dose vaccines (TIV-HD, hdQIV). The high-dose formulation boosts the immune response by delivering four times the standard antigen content (60 μg vs. 15 μg hemagglutinin per strain). Conversely, adjuvanted vaccines utilize MF59—an immune stimulant—to broaden the immune response and facilitate antigen sparing, achieving robust efficacy with a standard antigen dose [11].

Literature consistently demonstrates that these enhanced vaccines offer greater opportunities to reduce the influenza-related burden in older adults compared to traditional standard-dose vaccines [12,13,14]. Despite general consensus that older adults require vaccination, in many countries where different traditional standard-dose vaccines (including both aQIV and hdQIV) are available, recommendations issued by the competent authorities differ substantially, and even European public health recommendations lack uniformity (while many countries target individuals aged ≥65, others have lowered the threshold to ≥60 years) [15]. Moreover, the Italian national recommendation of use of any age-appropriate quadrivalent/trivalent traditional standard-dose vaccine for those aged ≥65 may streamline procurement but can lead to significant regional disparities in vaccination strategies [16,17].

Previous studies have largely compared enhanced vaccines against standard-dose non-adjuvanted vaccines [18,19].

Recent Health Technology Assessments (HTAs) in Italy have confirmed the safety, efficacy/effectiveness and economic value of aQIV [20,21]. However, these evaluations were mostly conducted through the prism of comparison with non-adjuvanted vaccines, which were considered standard of care. As healthcare systems move toward prioritizing enhanced vaccines for the elderly, understanding the relative vaccine effectiveness (rVE) between MF59-adjuvanted and high-dose formulations and between MF59-adjuvanted and standard-dose vaccines becomes essential for evidence-based policy making.

The objective of this systematic review is to explore and summarize the available evidence regarding the relative vaccine effectiveness (rVE) of enhanced influenza formulations—specifically MF59-adjuvanted—compared to high-dose (HD) vaccines and standard vaccines in adults aged ≥60 years.

Specifically, the analysis aims to determine the rVE of the MF59-adjuvanted influenza vaccine versus both non-adjuvanted standard-dose and non-adjuvanted high-dose influenza vaccines. Furthermore, this study seeks to assess how vaccine effectiveness varies across different age subgroups and in relation to the specific circulating influenza strains identified during the seasons under review, in order to provide a comprehensive overview of the protection offered by these enhanced formulations.

## 2. Methods

### 2.1. Reporting Standards

This systematic review was conducted in accordance with the Preferred Reporting Items for Systematic Review and Meta-Analyses (T) 2020 guidelines [22] (Table S1). The study protocol was developed a priori, shared within the research team and registered in the International Prospective Register of Systematic Reviews (PROSPERO; registration number: CRD420261407102). No amendments to the originally submitted protocol were made.

### 2.2. Eligibility Criteria

To define the inclusion criteria, the PICOS framework (Population, Intervention, Comparator, Outcome, Study design) was applied [22,23]. The criteria were specified as follows:

- Population (P): individuals aged ≥60 years;
- Intervention (I): aTIV/aQIV or hdTIV/hdQIV;
- Comparator (C): aTIV/aQIV or hdTIV/hdQIV or SD;
- Outcome (O): effectiveness;
- Study design (S): observational and pragmatic studies.

The study population comprised all older adults, as defined by the primary studies, hospital setting, risk profile (e.g., low or high risk) or other clinical or demographic characteristics.

Observational studies directly comparing aTIV/aQIV with hdTIV/hdQIV and aTIV/aQIV with SD (trivalent or quadrivalent) were considered eligible, as were pragmatic studies with the aforementioned outcomes. Trivalent (aTIV, hdTIV, SD) and quadrivalent (aQIV, hdQIV and SD) formulations were treated as interchangeable, given their shared manufacturing process and substantially overlapping compositions.

Outcomes included real-world effectiveness (*effectiveness*) across influenza-related endpoints (access to ED or hospitalization due to influenza). Broader medical-encounter, community influenza and influenza/pneumonia outcomes were identified separately; the hospital test-negative sensitivity analyses were performed.

Relative vaccine effectiveness (rVE) of aTIV/aQIV versus hdTIV/hdQIV was calculated, depending on study design, as (1 − relative risk [RR]) × 100%, (1 − hazard ratio [HR]) × 100% or (1 − odds ratio [OR]) × 100%, where RR, HR and OR represent statistically adjusted effect size (ES) measures.

Accordingly, negative rVE values (ES > 1) indicate lower effectiveness of aTIV/aQIV compared with hdTIV/hdQIV, whereas positive rVE values (ES < 1) indicate greater effectiveness. In line with recommendations [22], only statistically adjusted ES estimates were used in the evaluation of rVE.

The eligibility threshold of ≥60 years was specified a priori in the registered protocol and was chosen because the licensed indications of the two enhanced formulations under comparison are not aligned: MF59-adjuvanted vaccines (aTIV/aQIV) are authorized from 50 years of age in the EU and the UK following the 2024 extension of their marketing authorization, whereas high-dose vaccines (hdTIV/hdQIV) are authorized from 60 years of age [24,25,26,27]. National recommendations in Europe are correspondingly split between ≥60 and ≥65 years [15]. As reported in the following Section 3.1, every study meeting the eligibility criteria enrolled adults aged ≥65 years; all effectiveness estimates presented below consequently apply to that age group, and the absence of evidence for adults aged 60–64 years is reported as a finding of this review. This age-based population was considered irrespective of setting (e.g., community-dwelling, hospitalized or institutionalized), risk profile (e.g., low-risk vs. at-risk) or other clinical or demographic characteristics.

The primary endpoint of interest was hospitalization or presentation to the emergency department (ED) for influenza laboratory-confirmed or clinically diagnosed; the reconciled overall displays also retain the explicitly labeled broader endpoints described below. Exclusion criteria included non-original works (e.g., conference abstracts, editorials, books, systematic or narrative reviews, commentaries, expert opinions), clinical trials, no data for ages ≥60 and publications in languages other than English or Italian.

Hospital admission and presentation to the ED were combined into a single endpoint, as these are the two principal modes of contact between a patient with severe influenza and the healthcare system and the two that carry the greatest organizational and economic consequences for publicly funded health services. They also share the clinical feature that defines the construct of interest: both identify an episode severe enough to exceed the threshold for management in primary or ambulatory care.

### 2.3. Search Strategy and Selection Process

A comprehensive systematic search was performed combining automatic and manual approaches. The primary search was performed across the following databases: MEDLINE (via Ovid and PubMed). The search algorithm utilized a combination of Medical Subject Headings (MeSH) and free-text terms tailored to the PICO framework (e.g., fluad, MF59, high dose, efluelda, influenza vaccine, elderly, standard dose vaccine). The initial search was performed in late 2025 and updated prior to the final data analysis on 31 May 2026. Boolean operators (AND/OR) were applied to refine the output (Supplementary Table S2).

Additionally, a manual search was conducted using forward and backward citation tracking of included manuscripts via Google Scholar to capture any relevant gray literature. Articles obtained in the previous step were selected after reading the title and the abstract, evaluating them individually and discarding duplicate articles (two independent readers: D.A. and F.M.). The full text of the selected articles was read, and the most suitable publications were included.

Following the removal of duplicates using a semi-automatic method, the study selection proceeded in two stages. First, two independent reviewers (D.A. and I.S.) screened titles and abstracts to exclude ineligible records (i.e., those studies that were ineligible using the previously described PICOS algorithm). Second, the full texts of potentially eligible articles were reviewed in extenso. Eventual disagreements were resolved by consensus.

Data extraction was performed in duplicate using a predefined, pilot-tested Microsoft Excel spreadsheet. Extracted variables included: (i) full citation record; (ii) study location and influenza season(s); (iii) study design and data sources; (iv) participant characteristics (e.g., sample size); (v) vaccine types evaluated; (vi) predefined influenza-related outcomes.

### 2.4. Quality and Risk of Bias Assessment

The risk of bias (RoB) in the included non-randomized observational studies was independently assessed by two reviewers utilizing the Risk of Bias In Non-randomized Studies of Interventions (ROBINS-I) tool [28]. Studies were evaluated across seven distinct domains:

- Bias due to confounding;
- Bias in the selection of participants;
- Bias in the classification of interventions;
- Bias due to deviations from intended interventions;
- Bias due to missing data;
- Bias in measurement of outcomes;
- Bias in the selection of the reported results.

Based on this framework, studies were categorized as having a low, moderate, serious or critical risk of bias. Discrepancies were resolved via consensus. The RoB determined through the ROBINS-I tool was judged moderate in all included studies. In particular, all studies were at moderate RoB for the domains of residual confounding and reporting bias (Supplementary Tables S3 and S4).

### 2.5. Data Synthesis

Meta-analyses were conducted in R using the metafor package [29,30].

The main descriptive displays used inverse-variance random-effects models with the DerSimonian–Laird (DL) estimator on the log-ratio scale and normal-theory confidence intervals; sensitivity analyses examined alternative estimators and interval methods. For the overall comparisons in Section 3.3, the corrected selection retained one direct adjusted comparison per publication, prioritizing the influenza-related hospitalization or emergency department estimate when available. An overall estimate was preferred to its overlapping seasonal components, and hospital/ED composites were not counted alongside their nested inpatient components. These revisions are documented in Tables S5–S9. Because several of the included studies draw on very large national or commercial databases, the same individuals may have contributed to more than one publication, particularly where studies cover overlapping influenza seasons within the same country.

Broader medical-encounter, community influenza and influenza/pneumonia outcomes were retained where specified in the approved dataset. Age groups were analysed separately. For the broader-outcome adjuvanted-versus-high-dose age sensitivity analysis, the two influenza seasons described by Boikos et al. [31] were first combined within the study using fixed-effect inverse variance before the study-level estimates were pooled.

The one-estimate-per-publication rule described above applies to Section 3.3; dependence in the separately reported age and strain analyses is discussed in their respective sections. For the strain analysis, aggregate influenza A estimates were excluded when subtypes were reported.

Direct adjusted odds ratios separately by subtype or type were evaluated. We did not combine different subtypes because they share test-negative controls within publications and their adjusted-estimate covariances were unavailable [32]. Supplementary Table S7 reports exploratory within-subtype sensitivities, with one estimate per publication, using DL, REML and Paule–Mandel with normal, HK and modified HK intervals [33,34].

### 2.6. Effect Measures

Adjusted ORs, HRs and rate ratios were oriented as adjuvanted vaccine versus comparator, with ratios below 1 favoring the adjuvanted vaccine. Rate ratios from Poisson models were distinguished from cumulative risk ratios. A common logarithmic scale does not establish interchangeability [32]. Rare events may support some approximations, but rarity was not demonstrated for every extracted comparison; a single-season follow-up does not by itself equate an HR with a risk ratio. The interpretation of test-negative ORs depends on the sampling design and adjustment, and these estimates are not automatically exempt from non-collapsibility [34]. Consequently, the overall mixed-measure summaries are retained only as exploratory descriptive averages, not as estimates of a common causal risk ratio or a pooled percentage reduction in risk. We examined each native measure separately, omitted measure types in turn and restricted the standard-dose analysis to hospital test-negative ORs. We also examined published alternative models in the publications by Pott and Machado [35,36] without converting between measures. DerSimonian–Laird estimation and normal-theory intervals were held constant across these checks to isolate selection and measure choices.

Results and source provenance are provided in Tables S5 and S6. The estimator and interval comparisons in Supplementary Table S8 used the same corrected inputs and are reported separately from these selection checks.

### 2.7. Heterogeneity Assessment

Statistical heterogeneity was assessed using Cochran’s Q, the DerSimonian–Laird between-study variance estimate (τ^2^) and I^2^. A Q-test *p* value below 0.10 was treated as evidence of heterogeneity. Ninety-five per cent confidence and prediction intervals were calculated from the random-effects models.

Both are conditional on the model and independence assumptions; prediction intervals do not correct for dependence or for differences between effect measures. Prediction intervals and heterogeneity estimates are particularly unstable with few studies. The mixed-measure summaries and their intervals are interpreted as exploratory. A confidence interval describes uncertainty in the estimated average, whereas a prediction interval describes the model-based range for the underlying effect in a comparable new study.

Comparisons of DL, restricted maximum likelihood (REML) and Paule–Mandel estimators had already been conducted during preparation of the initial manuscript. We reproduced the archived results and updated these comparisons on the corrected selection. For each estimator, we separately calculated normal-theory, Hartung–Knapp (HK) and modified HK confidence intervals [30,33]. HK used a residual variance adjustment and a t distribution with k − 1 degrees of freedom; modified HK prevented the adjusted standard error from falling below the conventional standard error. K denotes the number of estimates in a model containing one contribution per publication, not proof of independent participants. Unmodified HK can give shorter intervals in some configurations, whereas modified HK can be very conservative with only two or three studies. Neither method corrects dependence, mixed-effect measures or bias. We treated small syntheses as descriptive and did not use statistical significance alone to judge robustness.

## 3. Results

### 3.1. Characteristics of Included Studies

**Figure 1:**
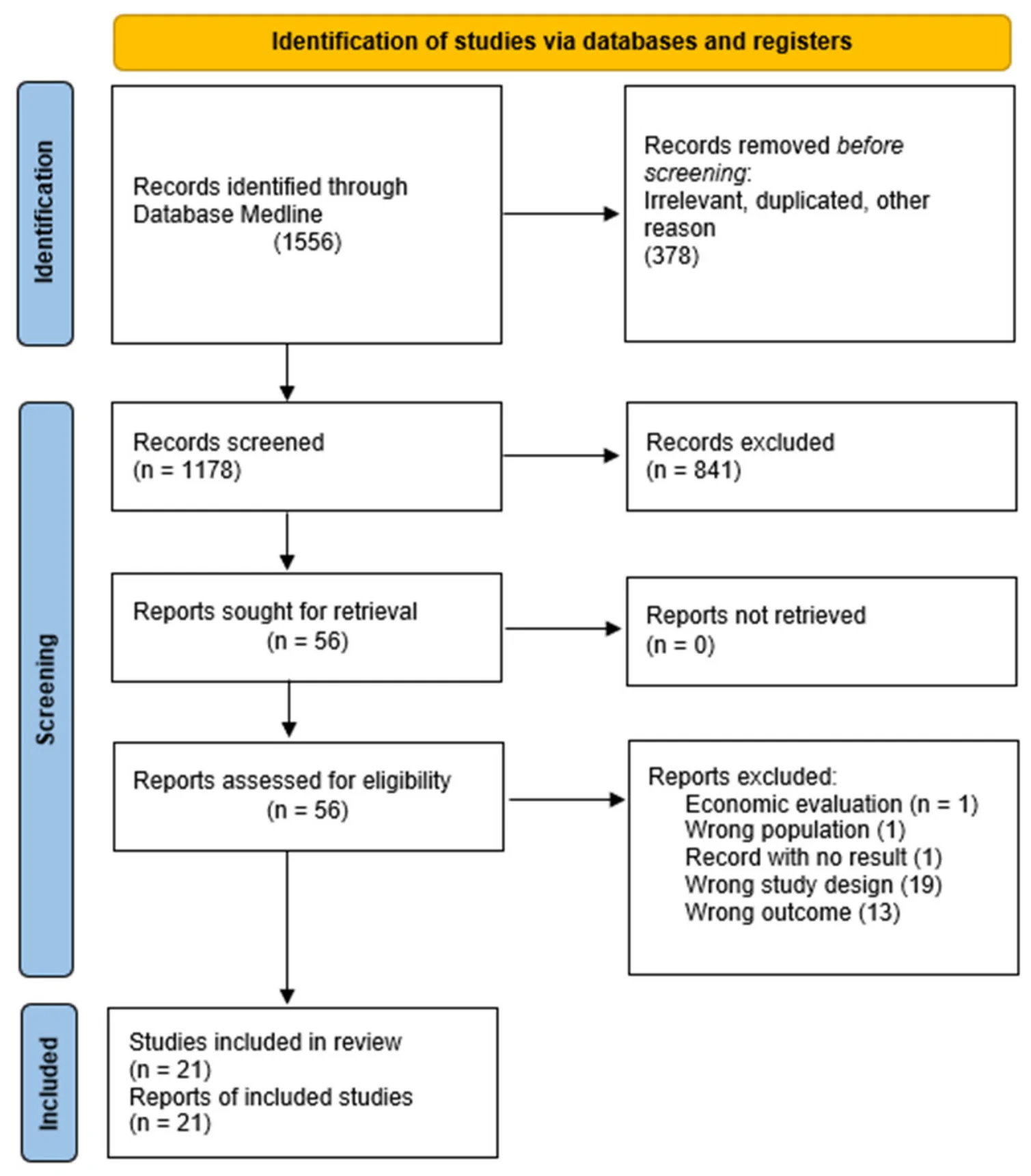
Flowchart of the study selection process.

The initial automatic search produced a total of 1556 records identified for screening by title and abstract. After removing duplicates (n = 378), 1122 were judged ineligible; 56 records were screened for full-text evaluation. Thirty-eight reports were excluded (economic evaluation, wrong population, record with no result, wrong study design and wrong outcome) (Figure 1). The reconciled dataset contained 13 publications comparing ADJ with HD: 12 provided overall-population estimates [12,31,35,37,38,39,40,41,42,43,44,45], while one (Imran et al., 2024) provided risk-stratum estimates only [46]. The characteristics of the included studies are reported in Table 1. Results were interpreted with consideration of study design and heterogeneity, rather than assuming equivalence across designs. For ADJ versus SD, eight publications were represented (Table 1) [31,36,45,47,48,49,50,51]. The available studies comparing ADJ vs. HD were conducted between 2016/17 and 2023/24 in Northern Hemisphere influenza seasons (all in the US). The studies that compared VE of ADJ vs. non-adjuvanted vaccines SD were performed in different countries: the US, the UK, Canada and Italy. The study population included immunocompetent adults aged ≥65 years with or without comorbidities. Several studies included multiple seasons. Additionally, a supplementary search was conducted in Google Scholar using forward and backward citation tracking of all included studies to identify potentially relevant records not indexed in MEDLINE. Four targeted searches were also performed using the following queries: “allintitle: adjuvanted influenza vaccine high-dose,” “allintitle: MF59 high-dose,” “allintitle: Fluad high-dose” and “allintitle: adjuvanted standard-dose influenza.” Overall, 57 records were identified and screened by title and abstract. Potentially relevant records were independently assessed by two reviewers (D.A. and F.M.), with duplicates removed before full-text assessment. The full texts of potentially eligible records were subsequently reviewed against the predefined eligibility criteria. No additional studies met the inclusion criteria and were therefore included in the systematic review.

### 3.2. Risk of Bias Assessment

Although some concerns were identified across specific domains of the Risk of Bias in observational studies (RoBINS-I) tool, the overall risk of bias for the outcome was judged to be low to moderate [28] (Tables S3 and S4).

### 3.3. Findings from Meta-Analysis

#### 3.3.1. Adjuvanted Vaccines Versus High-Dose Vaccines

**Figure 2:**
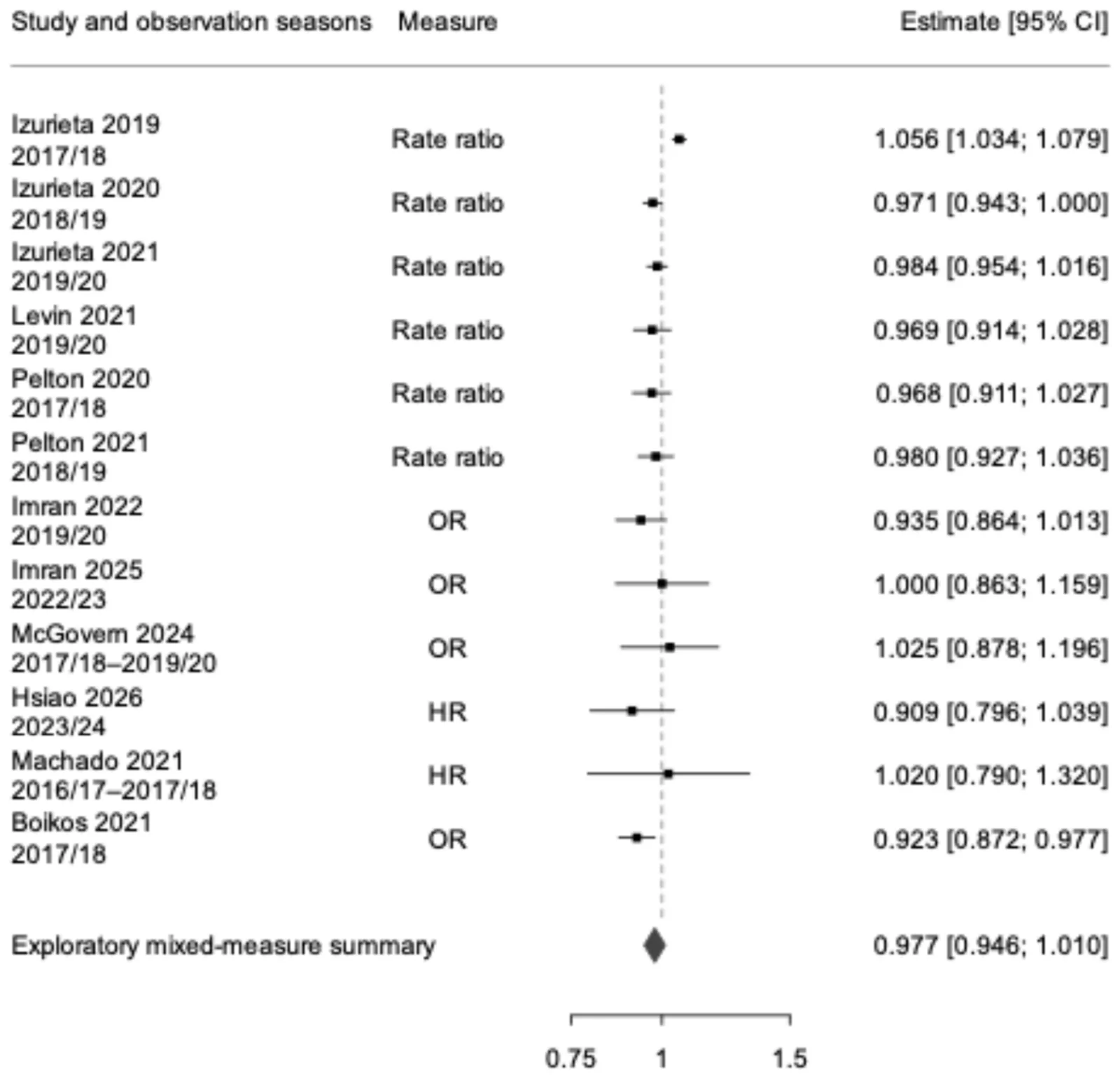
Adjusted estimates for MF59-adjuvanted versus high-dose influenza vaccines and an exploratory mixed-measure summary. Original measures and observation seasons are shown explicitly. One estimate is displayed per publication. Equal marker sizes do not imply independent populations. The summary assumes independent rows and is not a common causal risk ratio. Data from references [12,31,35,37,38,39,40,41,42,43,44,45].

The overall reference display comprised 12 adjusted estimates from 12 publications: six rate ratios, four ORs and two HRs (Figure 2) [12,31,35,37,38,39,40,41,42,43,44,45]. Each publication contributed once. An influenza hospital/ED estimate was selected where available; Boikos retained its broader medical-encounter endpoint, and nested inpatient estimates and overlapping seasonal estimates were not additionally included. Imran et al. (2024) [46] was evaluated separately in two sensitivity scenarios because its approved estimates were specific to risk strata rather than the overall population.

The displayed summary uses DL with normal-theory intervals; alternative intervals are in Supplementary Table S8. The exploratory mixed-measure summary was 0.977 (95% CI 0.946 to 1.010). Heterogeneity remained substantial (Q = 45.5, df = 11, I^2^ = 75.8%; τ^2^ = 0.00195). The conditional 95% prediction interval was 0.891 to 1.072. This summary is not expressed as a pooled rVE because its constituent measures have different interpretations.

The overall reference summary did not establish a clear average difference between the two enhanced vaccines; results varied across measure-specific subsets. These findings do not establish equivalence. Participant overlap across claims, databases or seasons remains possible and cannot be eliminated by selecting one estimate per publication.

#### 3.3.2. Adjuvanted Vaccines Versus Non-Adjuvanted Vaccines (Standard Dose)

**Figure 3:**
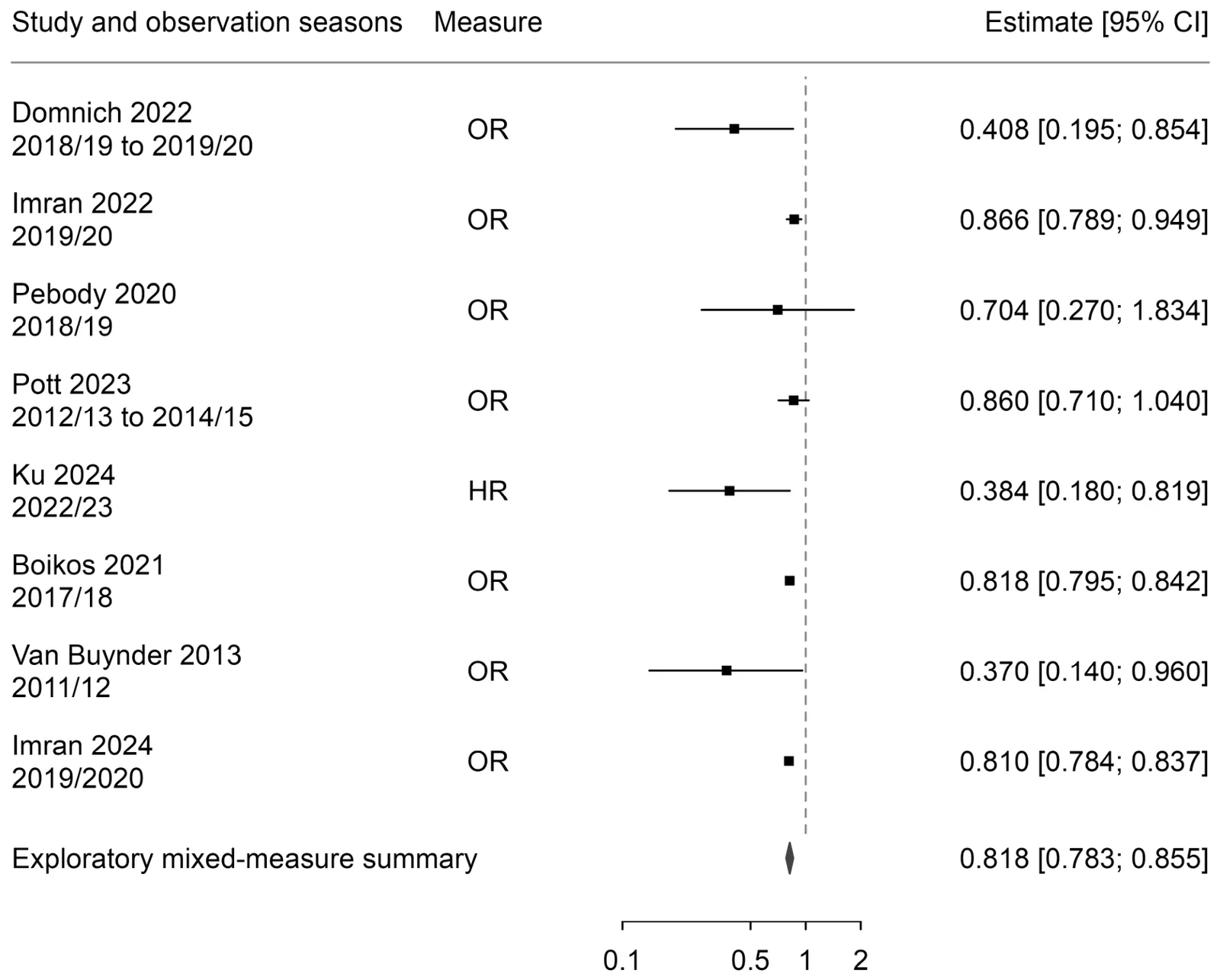
Adjusted estimates for MF59-adjuvanted versus standard-dose influenza vaccines and an exploratory mixed-measure summary including explicitly labeled broader outcomes. Source measures and seasons are displayed explicitly. One estimate is displayed per publication; the summary is conditional on independence and is not a common causal risk ratio. Data from references [31,36,45,47,48,49,50,51]. The displayed summary uses DL with normal-theory intervals; alternative intervals are in Supplementary Table S8.

The overall reference display comprised eight estimates from eight publications: seven ORs and one HR (Figure 3) [31,36,45,47,48,49,50,51].

Pott et al. contributed the direct comparative odds ratio (OR) rather than the absolute effectiveness estimate versus unvaccinated participants; the frailty-adjusted model of the same publication was assessed separately [35]. Domnich et al. contributed the estimate for any influenza, without incorporating the additional estimates for influenza A or specific subtypes [49]. The eligible inpatient/emergency department (ED) outcome based on admitting diagnosis was obtained from Imran et al. [45], which was already represented in the extraction dataset. Van Buynder contributed community laboratory-confirmed influenza, Boikos contributed broader medical encounters, and Imran et al. (2024) contributed influenza/pneumonia hospitalization among adults with at least one high-risk condition [31,47,50]. These outcomes were identified explicitly, and the existing hospital test-negative analyses were retained separately.

The exploratory mixed-measure summary was 0.818 (95% CI 0.783 to 0.855). It is not interpreted as a pooled percentage reduction in risk. The individual source estimates retain their original OR or HR interpretation.

Heterogeneity was estimated at I^2^ = 41.6% (Q = 12.0, df = 7; τ^2^ = 0.00102). The conditional 95% prediction interval was 0.758 to 0.883 under DL with normal-theory intervals. The favorable estimated average does not establish a consistent advantage in a comparable new setting. The sensitivity analyses below show that the magnitude and uncertainty also depend on study selection, adjustment and the interval method. For the same DL estimate, both HK and modified HK gave a 95% confidence interval of 0.766 to 0.875, which excludes 1; the Paule–Mandel modified-HK prediction interval, however, was 0.422 to 1.317.

#### 3.3.3. Sensitivity Analyses

Supplementary Table S5 reports the sensitivity analyses. Against high-dose vaccines, estimates restricted to rate ratios, ORs and HRs were 0.990 (95% CI 0.953 to 1.029), 0.940 (95% CI 0.901 to 0.981) and 0.931 (95% CI 0.828 to 1.048), respectively. The OR-only subset and the subset omitting rate ratios had normal-theory intervals below 1; other measure-omission intervals included 1. These are conditional comparisons of different study sets, not tests of interaction by measure.

Against standard-dose vaccines, the seven-OR summary was 0.819 (95% CI 0.792 to 0.847); the single HR was reported individually. Restriction to the three hospital test-negative ORs gave 0.693 (0.437 to 1.099) with results from Pott et al. and 0.681 (0.503 to 0.923) with its additional frailty adjustment [35]. The point estimates were close, but whether the interval included 1 changed. Replacing Machado’s Cox HR with its published Poisson rate ratio had little effect on the high-dose descriptive summary. These results support cautious interpretation rather than an assumption that all measures or conclusions are interchangeable.

The updated estimator and interval comparisons are reported in Supplementary Table S8. Against high-dose vaccines, the DL, REML and Paule–Mandel estimates were 0.977, 0.979 and 0.981, respectively; all normal-theory, HK and modified HK intervals included 1. Against standard-dose vaccines, the corresponding estimates were 0.818, 0.816 and 0.746. Their normal-theory, HK and modified HK intervals excluded 1; modified HK intervals were 0.766 to 0.875, 0.790 to 0.844 and 0.586 to 0.948, respectively. The Paule–Mandel modified-HK prediction interval included 1 (0.422 to 1.317). Among the three hospitals’ test-negative studies, both Pott adjustment choices yielded HK and modified HK intervals that included 1 under all three estimators. Thus, the favorable average in the eight-publication SD display did not imply a consistent advantage across settings or analytical subsets.

### 3.4. Age Groups

Age-stratified analyses were conducted separately for ages 65–74, 75–84 and 85 years or older. They were exploratory because the common-endpoint adjuvanted-versus-high-dose models contained only two publications and two estimates per age group, while each adjuvanted-versus-standard-dose model contained four estimates from three publications. None of these models included more than three publications, and the pooled values should therefore be read as descriptive summaries of the individual estimates rather than as stable effect sizes.

#### 3.4.1. Adjuvanted Vaccines Versus High-Dose Vaccines in Different Age Groups

**Figure 4:**
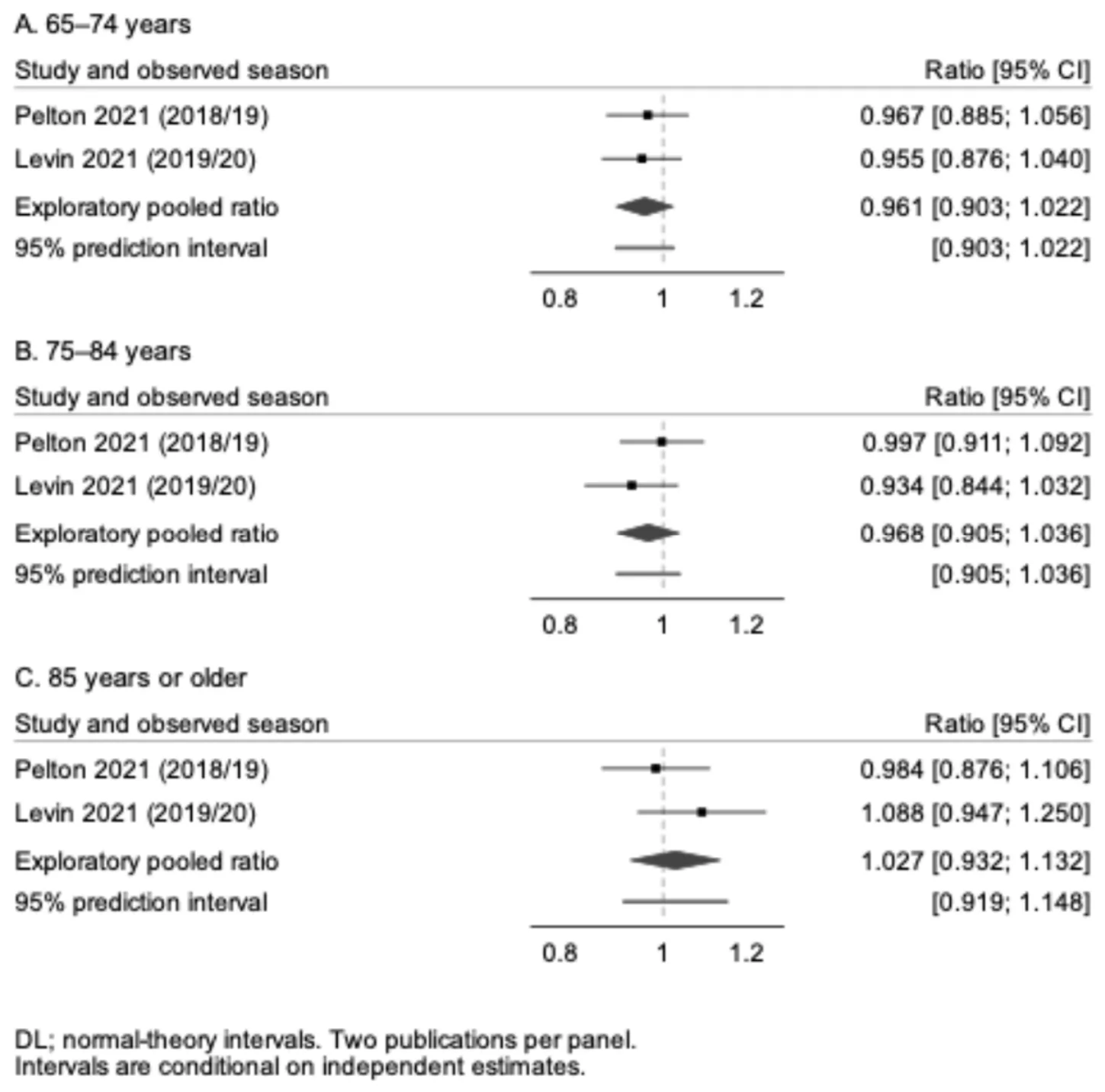
Random-effects meta-analyses of MF59-adjuvanted versus high-dose vaccines by age group: (**A**) 65–74 years, (**B**) 75–84 years and (**C**) 85 years or older. Data from Pelton et al. [40] and Levin et al. [41].

Three studies reported age-stratified estimates for adults aged 65–74, 75–84 and ≥85 years: Pelton et al. [40], Boikos et al. [31] and Levin et al. [41]. Pelton and Levin evaluated influenza-related hospitalizations or emergency-department visits and were combined in the primary subgroup meta-analysis. Boikos evaluated any influenza-related medical encounter and was therefore excluded from the primary pooling because the outcome was broader. The results of these analyses are shown in Figure 4.

Each primary common-endpoint analysis included two publications and two estimates. Pooled ratios were 0.961 (95% CI 0.903 to 1.022; I^2^ = 0%) for ages 65–74 years, 0.968 (95% CI 0.905 to 1.036; I^2^ = 0%) for ages 75–84 years and 1.027 (95% CI 0.932 to 1.132; I^2^ = 15.3%) for ages 85 years or older. None of the confidence intervals excluded 1, and the small number of publications limits inference. Modified HK intervals under DL were 0.645 to 1.432, 0.626 to 1.498 and 0.546 to 1.931, respectively (Supplementary Table S8). These conditional two-publication calculations are highly imprecise; a zero heterogeneity estimate does not establish homogeneity.

In an exploratory broader-outcome sensitivity analysis, the two Boikos seasons were first combined within study by fixed-effect inverse variance, and the resulting three study-level estimates were pooled using the DerSimonian–Laird random-effects model. The pooled ratios were 0.943 (95% CI 0.908 to 0.980; I^2^ = 0%) for ages 65–74 years, 0.937 (95% CI 0.881 to 0.996; I^2^ = 40.1%) for ages 75–84 years and 0.980 (95% CI 0.884 to 1.088; I^2^ = 56.4%) for ages 85 years or older. This sensitivity analysis combines a broader medical-encounter outcome with hospitalization or emergency department outcomes and should not replace the common-endpoint analysis. The corresponding modified HK intervals under DL were 0.867 to 1.025, 0.818 to 1.072 and 0.778 to 1.235, all including 1 (Supplementary Table S8). The within-publication seasonal aggregation also assumes independence and does not resolve possible participant overlap.

#### 3.4.2. Adjuvanted Vaccines Versus Standard-Dose Vaccines in Different Age Groups

Pott et al. [35], Boikos et al. [31] and Imran et al. [45] contributed four estimates to each age-group analysis. Multiple Boikos seasons were analyzed as separate estimates, so within-publication correlation was not modeled. Pebody age estimates compared vaccination with no vaccination and were excluded after source verification and author confirmation; its direct overall and subtype comparisons were retained.

**Figure 5:**
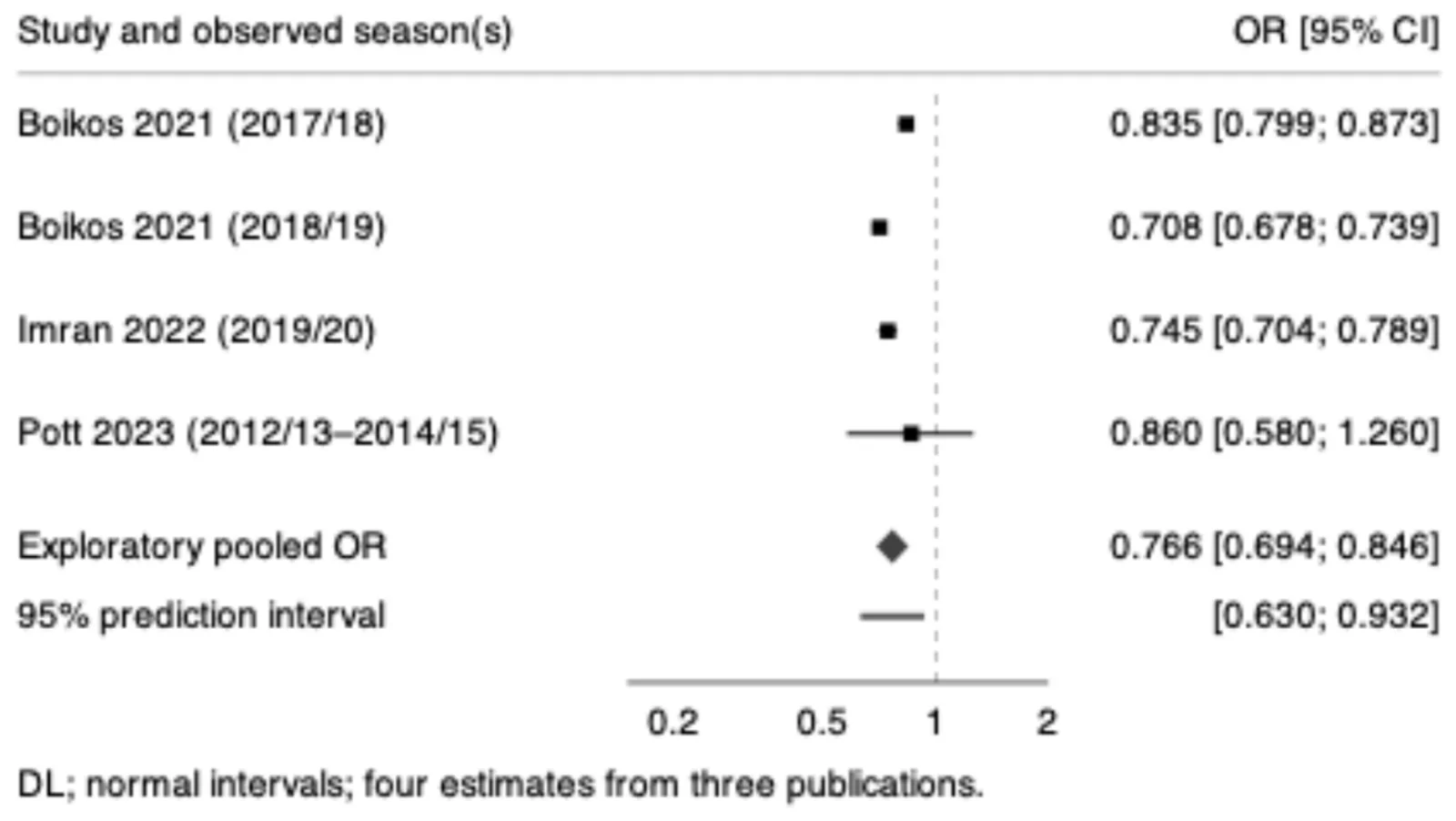
Random-effects meta-analysis of MF59-adjuvanted versus standard-dose vaccines among adults aged 65–74 years. Data from Pott et al. [35], Boikos et al. [31] and Imran et al. [45].

For ages 65–74 years (Figure 5), the pooled OR was 0.766 (95% CI 0.694 to 0.846; *p* < 0.001). The 95% prediction interval was 0.630 to 0.932.

Heterogeneity was very high (Q = 28.6, df = 3, *p* < 0.001; I^2^ = 89.5%), so the pooled estimate should be interpreted as an average across heterogeneous estimates.

**Figure 6:**
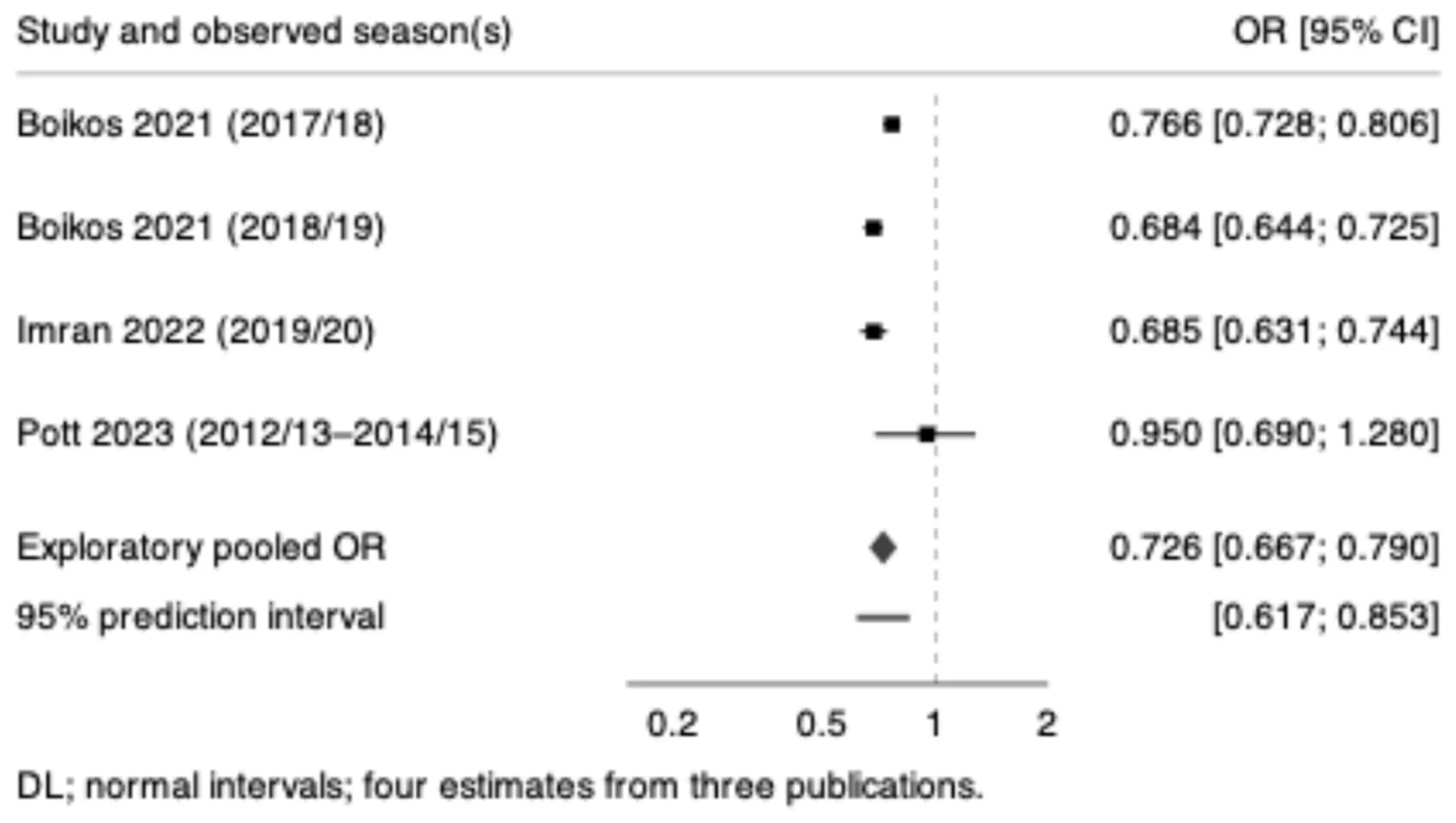
Random-effects meta-analysis of MF59-adjuvanted versus standard-dose vaccines among adults aged 75–84 years. Data from Pott et al. [35], Boikos et al. [31] and Imran et al. [45].

For ages 75–84 years (Figure 6), the same three publications contributed four estimates.

The pooled OR was 0.726 (95% CI 0.667 to 0.790; *p* < 0.001). The 95% prediction interval was 0.617 to 0.853.

Heterogeneity remained substantial (Q = 13.0, df = 3, *p* = 0.005; I^2^ = 76.9%).

**Figure 7:**
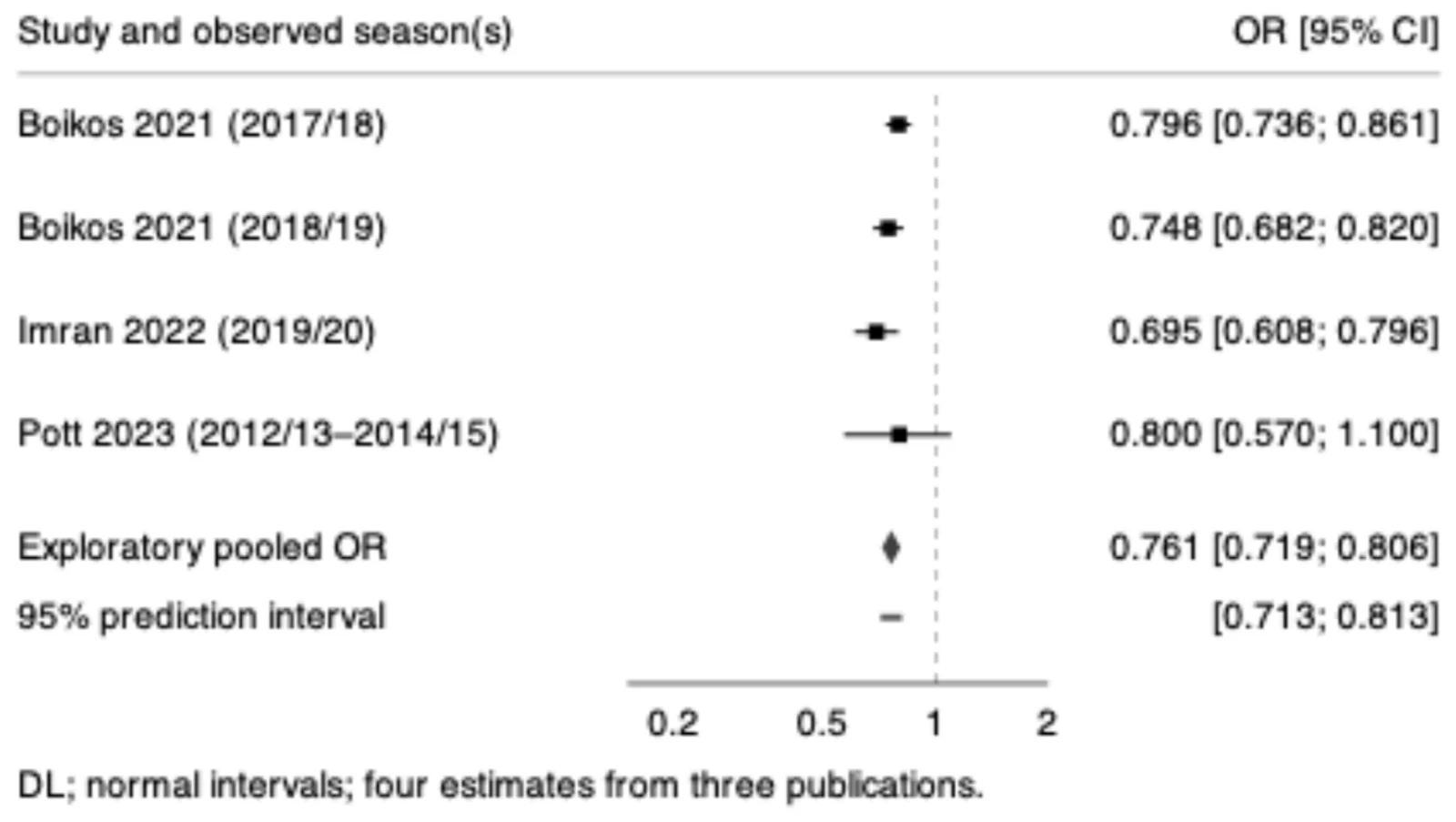
Random-effects meta-analysis of MF59-adjuvanted versus standard-dose vaccines among adults aged 85 years or older. Data from Pott et al. [35], Boikos et al. [31] and Imran et al. [45].

For adults aged 85 years or older (Figure 7), the same three publications contributed four estimates.

The pooled OR was 0.761 (95% CI 0.719 to 0.806; *p* < 0.001). The 95% prediction interval was 0.713 to 0.813.

Heterogeneity was low (Q = 3.2, df = 3, *p* = 0.359; I^2^ = 6.9%).

All three pooled ratios favored the adjuvanted vaccine, but the analyses were based on only three publications, included correlated seasonal estimates and did not formally test differences between age groups. They should therefore be considered exploratory. Their reported intervals are conditional on treating rows as independent; substituting another heterogeneity estimator or counting the four rows as four independent studies for HK would not validate that assumption.

### 3.5. Strains

Pott et al. [35], Domnich et al. [49] and Pebody et al. [48] reported direct estimate comparisons of MF59-adjuvanted and standard-dose vaccines for laboratory-confirmed influenza-associated hospitalization.

**Figure 8:**
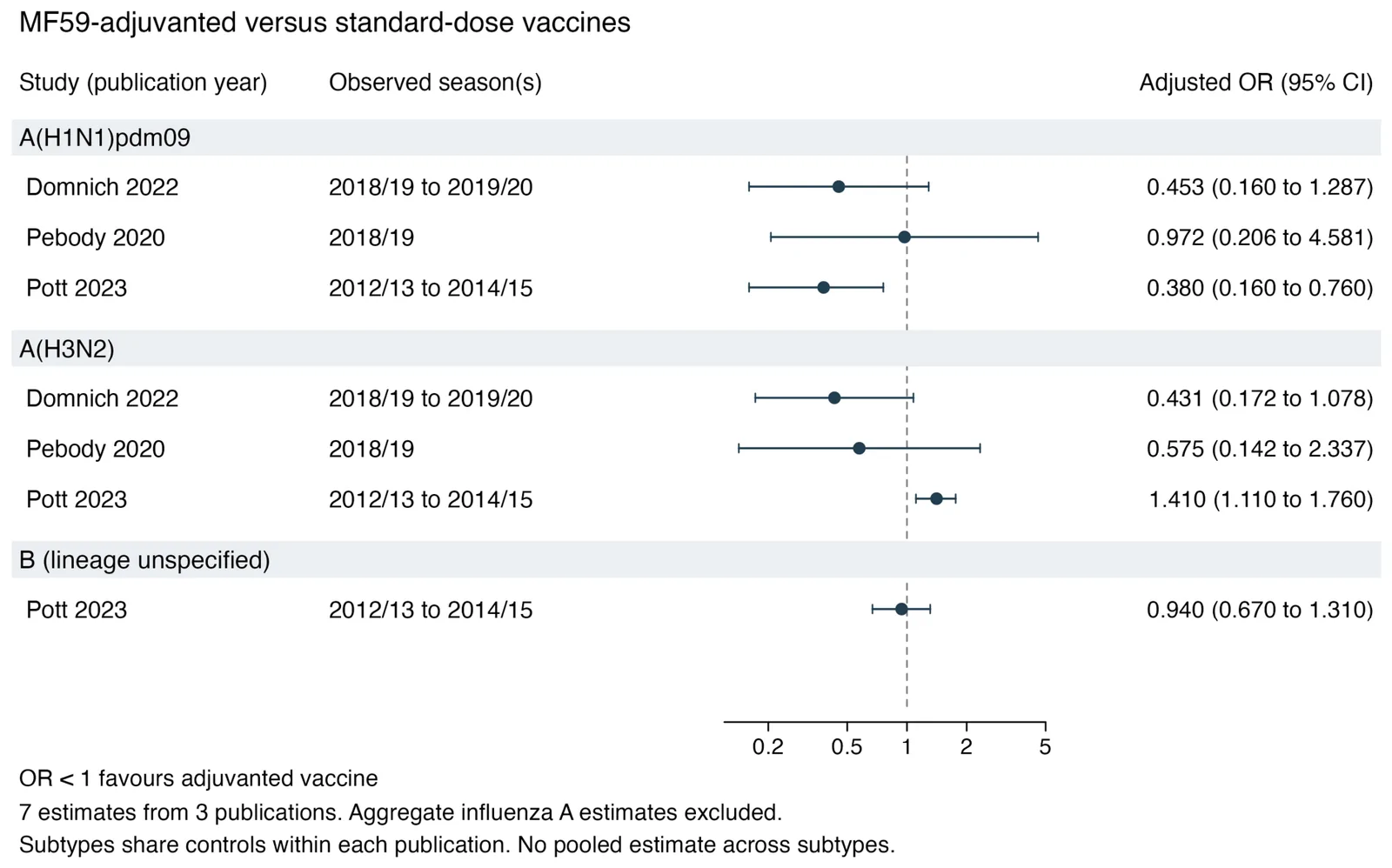
Direct adjusted odds ratios for MF59-adjuvanted versus standard-dose vaccines by influenza subtype or type. Points and 95% confidence intervals are the published comparative estimates, expressed as odds ratios. Aggregate influenza A estimates are excluded; no summary across subtypes is shown. Publication years and observed seasons are identified separately: Pebody, 2018/19 [48]; Domnich, 2018/19 to 2019/20 [49]; Pott, 2012/13 to 2014/15 [35]. Data from Pott et al. are examined in Supplementary Table S7C.

We excluded the aggregate influenza A estimates from Pott and Domnich because they overlap the separately reported A(H1N1)pdm09 and A(H3N2) cases. Figure 8 therefore contains seven estimates from three hospital test-negative studies: three for each influenza A subtype and one for influenza B. Source values, comparators and observed seasons are documented in Supplementary Table S7A.

### 3.6. Matching Vaccine Strains

**Figure 9:**
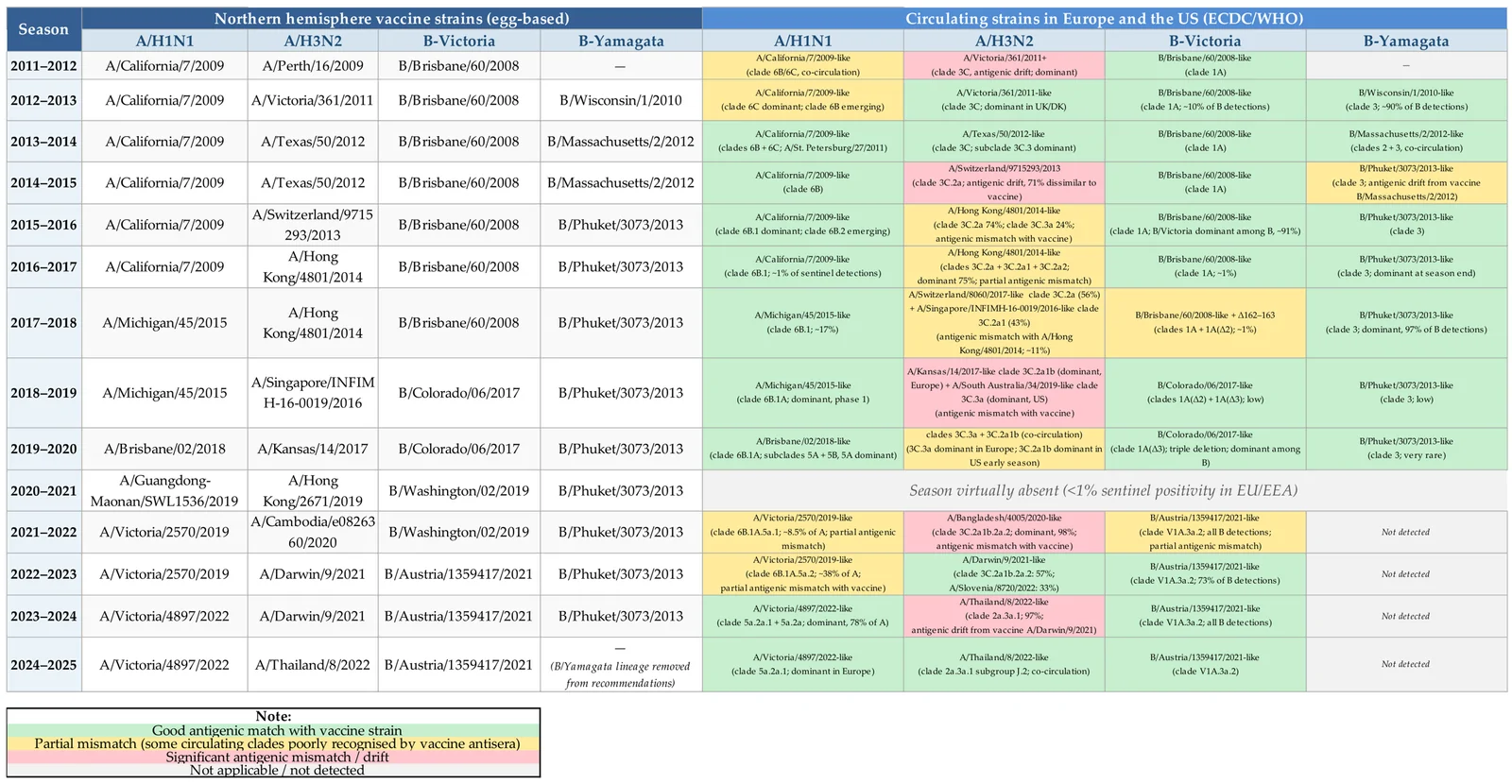
Influenza vaccine strain composition for the Northern Hemisphere and the corresponding circulating influenza virus strains during the 2011/2012–2024/2025 influenza seasons. Data from WHO and CDC/ECDC [52,53].

The vaccine strain composition for the Northern Hemisphere and the circulating strains from 2011/12 to 2024/25 influenza seasons is depicted in Figure 9, where data highlight the substantial temporal variability of influenza viruses and the resulting need for periodic vaccine updates.

Figure 8 displays direct adjusted odds ratios separately by subtype or type. We did not combine different subtypes because they share test-negative controls within publications and their adjusted-estimate covariances were unavailable [32]. Supplementary Table S7 reports exploratory within-subtype sensitivities, with one estimate per publication, using DL, REML and Paule–Mandel with normal, HK and modified HK intervals [30,33].

The estimates are presented descriptively by subtype or type in Figure 8. Removal of aggregate influenza A avoids duplicate cases but does not remove the dependence caused by shared test-negative controls across subtypes within each study. We therefore report no pooled effect across subtypes. Exploratory within-subtype analyses used one estimate from each of the three publications (Supplementary Table S7B,C). With Pott results, DL gave an OR of 0.457 for A(H1N1)pdm09 (95% modified HK CI 0.128 to 1.630) and 0.794 for A(H3N2) (0.111 to 5.669). All HK and modified HK intervals included 1 under DL, REML and Paule–Mandel, including the alternative analysis using Pott’s additional frailty adjustment. With three studies per subtype, heterogeneity and uncertainty are poorly determined; these results do not establish a consistent subtype-specific advantage.

Individual estimates varied. In Pott et al., the results (Supplementary Table S7C) show that the A(H1N1)pdm09 OR was 0.38 (95% CI 0.16 to 0.76), whereas the A(H3N2) OR was 1.41 (1.11 to 1.76). With additional frailty adjustment, the latter became 1.13 (0.88 to 1.44), illustrating sensitivity to adjustment [35]. We corrected the two Pebody inputs to the direct vaccine comparisons: OR 0.972 (0.206 to 4.581) for A(H1N1)pdm09 and 0.575 (0.142 to 2.337) for A(H3N2); both intervals include 1. The single influenza B estimate from Pott was 0.94 (0.67 to 1.31). The source does not identify a lineage for this estimate, so it cannot be interpreted as B/Victoria-specific. No formal comparison between subtypes was made because their within-study covariance was unavailable.

## 4. Discussion

Influenza vaccination remains the cornerstone of influenza prevention and is the most effective public health intervention for reducing influenza-associated morbidity, hospitalizations and mortality, particularly among older adults and individuals with underlying chronic medical conditions. However, VE varies considerably between influenza seasons and across population groups [1,2]. This variability is primarily driven by antigenic drift, whereby the influenza virus accumulates mutations in surface glycoproteins that reduce the antigenic match between circulating strains and vaccine components. In addition, the production of most seasonal influenza vaccines in embryonated chicken eggs can introduce adaptive mutations, particularly in the hemagglutinin protein of A(H3N2) viruses, leading to egg-adapted antigenic changes that may further compromise vaccine-induced immune responses. Other factors influencing VE include the recipient’s age, immune status, prior vaccination history and the type of vaccine administered. Consequently, enhancing vaccine performance through the development of improved vaccine formulations, such as adjuvanted, high-dose, recombinant and cell-based vaccines, has become a major priority for protecting populations at increased risk of severe influenza outcomes [54]. Over the past decades, the evolving landscape of influenza vaccination has led to the development and global introduction of improved vaccine formulations within immunization programs. These enhanced vaccines aim to improve protection, particularly among older adults and other populations with suboptimal immune responses [8,24,55,56]. They have been specifically designed to address the limitations of conventional standard-dose, egg-based inactivated influenza vaccines by eliciting broader and more robust immune responses. Currently available enhanced influenza vaccines include MF59-adjuvanted inactivated influenza vaccines (aIIV), high-dose inactivated influenza vaccines (HD-IIV), recombinant influenza vaccines (RIV) and cell-culture-derived influenza vaccines (ccIIV) [26]. Each formulation employs a distinct strategy to improve immunogenicity: adjuvanted vaccines enhance the magnitude and breadth of the immune response through the MF59 oil-in-water emulsion, whereas high-dose vaccines increase antigen content to elicit stronger immunogenicity [26].

Vaccine effectiveness is typically assessed by comparing health outcomes between vaccinated and unvaccinated individuals or by evaluating new vaccine formulations against existing standard vaccines. Several factors have been proposed to explain the suboptimal performance of influenza vaccines, including antigenic drift, original antigenic sin, pre-existing immune imprinting and incomplete induction of broadly neutralizing antibodies.

Our systematic review provides a comprehensive and up-to-date evaluation of the rVE of the MF59-adjuvanted influenza vaccine compared with high-dose and standard-dose vaccines. Eligibility was set a priori at ≥60 years because the MF59-adjuvanted vaccine (aTIV/aQIV) had its licence extended in 2024 from ≥65 years to include adults aged 50–64 years in the UK and the EU [26,27], whereas the high-dose vaccine is licensed from ≥60 years; a narrower a priori threshold would have precluded the identification of any evidence in the younger age band. No study meeting the eligibility criteria enrolled adults below 65 years of age, and every estimate reported here therefore applies to adults aged ≥65 years. The absence of comparative effectiveness data for adults aged 60–64 years is itself relevant to countries considering whether to extend the use of enhanced vaccines below 65 years and represents a priority for future research.

In the corrected overall comparisons, the mixed-measure summary against high-dose vaccines was close to 1 and did not establish equivalence. Against standard-dose vaccines, the exploratory average favored the adjuvanted vaccine, but the conditional Paule–Mandel modified-HK 95% prediction interval (0.422 to 1.317) included 1. The DL 95% HK confidence interval for the average (0.766 to 0.875) excluded 1, whereas HK intervals in the three-study hospital test-negative subset included 1. A consistent advantage across settings was therefore not established; restricting the analysis by design or changing the adjustment set further affected its magnitude or uncertainty. These exploratory averages do not identify a single causal risk reduction across all designs and settings.

Previous systematic reviews have addressed the comparative effectiveness of MF59-adjuvanted and high-dose vaccines, and our findings are broadly consistent with them. For example, Domnich et al. [14] included 10 retrospective cohort studies (US, 2016–2020 seasons) with outcomes on influenza-related hospitalization and demonstrated that MF59-adjuvanted standard-dose vaccine and high-dose vaccine showed comparable effectiveness with small differences in some analyses that were inconsistent and often depended on outcome definition or study sponsorship. Coleman BL et al. [57] investigated twenty-one studies conducted during the 2006/07–2019/20 influenza seasons in their qualitative review; 16 studies were meta-analyzed. aTIV reduced medical encounters due to lab-confirmed influenza with pooled estimates of 58.5% (40.7, 70.9; I^2^ = 52.9%) for hospitalized patients. The pooled estimates for the relative VE of aTIV for the prevention of influenza-related medical encounters were 13.9% (4.2, 23.5; I^2^ = 95.9%) compared with TIV, 13.7% (3.1, 24.2; I^2^ = 98.8%) compared with QIV and 2.8% (−2.9, 8.5; I^2^ = 94.5%) compared with HD-TIV. Among adults ≥ 65 years, aTIV demonstrated significant absolute VE, improved relative VE compared to non-adjuvanted standard-dose TIV/QIV and comparable relative VE to high-dose TIV.

Our corrected descriptive summaries show a similar direction of association, while the standard-dose sensitivity analyses qualify its certainty. The present review adds age-stratified and strain-specific analyses and an explicit quantification of the uncertainty attached to each pooled estimate.

Effect modification by subtype and by age has been proposed as an explanation for the variability of influenza VE across seasons. Guo et al. [58] found that vaccine effectiveness varied by influenza virus subtype and age group, with lower protection against A(H3N2) and among older adults, and concluded that more effective vaccination strategies are needed in these populations.

These findings bear on current vaccination policy for older adults. National advisory bodies in the United States and in Canada already recommend enhanced influenza vaccines—specifically MF59-adjuvanted (aQIV) and high-dose (QIV-HD) formulations—in preference to standard-dose vaccines for adults aged ≥65 years: the Advisory Committee on Immunization Practices lists both among the preferred vaccine types for this age group [56], and the National Advisory Committee on Immunization recommends their preferential use [59].

Our estimates generally favor adjuvanted vaccines over standard-dose vaccines, but the sensitivity analyses and possible dependence limit the strength of this comparison. They do not, however, support a preference between the two enhanced formulations, nor do they support a claim of uniform superiority over standard-dose vaccines across settings: the conditional Paule–Mandel modified-HK 95% prediction interval for the latter comparison (0.422 to 1.317) spans both sides of the null. Both the presence and magnitude of an advantage in a particular season and setting remain uncertain; inclusion of the null does not establish equivalence.

In our systematic review, outcomes included hospital admissions and emergency department visits for laboratory-confirmed or clinically diagnosed influenza, as well as explicitly identified broader medical-encounter, community influenza and influenza/pneumonia endpoints. Indeed, the protection induced by influenza vaccines can be measured against a range of influenza-related outcomes. However, laboratory-confirmed influenza (LCI) can be considered the “gold standard,” as it is the most specific outcome according to the World Health Organization (WHO) [60].

It is important to note that influenza diagnoses identified through coded data in administrative databases—such as hospital discharge or admission records—can be considered highly specific. Prior research has demonstrated strong concordance between ICD-10 influenza diagnostic codes and laboratory-confirmed influenza. In particular, studies comparing ICD-10 coding with PCR results have reported specificity estimates of approximately 98–99% for influenza-specific codes assigned at hospital discharge [61,62]. Given this high specificity, both influenza-coded events in administrative datasets and influenza-related complications were considered primary outcomes in this analysis.

### Strengths and Limitations

The primary strength of this work lies in its timeliness and comprehensive update of the available evidence, incorporating the most recent studies and providing an updated comparative assessment of MF59-adjuvanted, high-dose and standard-dose influenza vaccines in older adults. This review offers a detailed evaluation of vaccine performance across different older adult populations, considering relevant age subgroups and the evolving epidemiological characteristics of influenza. In particular, it reports vaccine effectiveness estimates by age category, including adults aged ≥65 years. Furthermore, the analysis considers the impact of circulating influenza strains and vaccine formulations, including estimates observed across influenza A subtypes (A/H1N1 and A/H3N2) and influenza B, without an identified lineage. By integrating evidence across multiple vaccine platforms, age groups and seasonal influenza strains, this work provides a comprehensive perspective on the potential role of enhanced vaccines in reducing the influenza-related disease burden among older adults.

A key limitation of systematic reviews is the heterogeneity of the included studies, arising from differences in methodological approaches, clinical characteristics, study populations and outcome definitions, which may contribute to variability in effect estimates. In the context of seasonal influenza, additional sources of variability include viral factors, such as antigenic drift, and vaccine-related factors, including egg adaptation, which may reduce the antigenic match between circulating strains and vaccine components. Host-related factors, including comorbidities, immune imprinting and immunosenescence, may further influence vaccine effectiveness. Although pooling data across multiple influenza seasons may increase heterogeneity due to differences in circulating strains and vaccine match, it provides an estimate of the average effect across diverse epidemiological contexts. Despite efforts to ensure comparability among studies regarding design, comparators and outcomes, substantial heterogeneity remained, particularly among cohort studies. Regarding the outcome, we combined hospitalization and emergency-department (ED) encounters into a composite outcome because both represent clinically important healthcare utilization resulting from acute deterioration and may capture different levels of the same underlying disease burden. Hospitalization reflects more severe events requiring inpatient management, whereas ED encounters capture acute episodes that warrant urgent medical evaluation but may not require admission. Thus, restricting the outcome to hospitalization alone could underestimate the overall burden of clinically significant acute events, particularly when an intervention may prevent an episode from progressing to hospitalization but still result in an ED visit.

The strain-specific evidence comprised only three publications, with shared test-negative controls across subtypes within each publication. We therefore displayed the individual estimates and restricted exploratory pooling to separate within-subtype analyses. The small number of studies and sensitivity to adjustment limited inference, and neither subtype effect modification nor lineage-specific influenza B effectiveness could be established.

Among the observational studies, assessment of study-level characteristics suggested potential variability in effect estimates, although formal interpretation is constrained by the limited number of studies available for certain comparisons. In addition, no studies comparing recombinant influenza vaccines and cell-culture-derived influenza vaccines were included.

A common log-ratio scale does not remove differences between ORs, HRs and rate ratios. The supplementary analyses examine the consequences of these choices but cannot isolate measure type from differences in study design, population or endpoint. Selecting one estimate per publication removes explicit within-publication duplication in the overall comparisons, but cross-publication overlap remains possible. Both confidence and prediction intervals may therefore misrepresent uncertainty, and prediction intervals do not remedy this limitation. Summaries based on few studies require particular caution. In addition, the overall standard-dose average excluded the null with HK and modified HK under DL, REML and Paule–Mandel, but the Paule–Mandel prediction interval and the hospital test-negative HK intervals included 1. With very few studies, even these interval methods and the prediction interval remain unstable; they cannot compensate for unmodeled dependence.

## 5. Conclusions

In adults aged ≥65 years, the exploratory overall comparison did not establish a clear difference between MF59-adjuvanted and high-dose vaccines. Against standard-dose vaccines, the exploratory average favored the adjuvanted vaccine, but the conditional Paule–Mandel modified-HK 95% prediction interval (0.422 to 1.317) included 1; these data do not establish a consistent advantage across settings. The overall standard-dose HK and modified HK confidence intervals excluded the null under all three estimators, whereas the three-study hospital test-negative analyses remained imprecise. These findings do not establish equivalence between vaccines. Mixed-effect measures, heterogeneity and potentially overlapping source populations limit causal and setting-specific conclusions. No eligible study reported estimates for adults aged 60–64 years, and comparative effectiveness in that age band remains to be established.

## Figures and Tables

**Table 1:** Characteristics of included studies estimating the relative vaccine effectiveness of adjuvanted influenza vaccines and non-adjuvanted high-dose vaccines and standard-dose vaccines in older adults aged ≥60 years.

Adjuvanted Vaccines Versus High-Dose Vaccines							
Authors	Study Design	Country	Influenza Season(s)	Study Population	Primary Influenza-Related Outcome(s)	N aTIV Users	N HDTIV/HDQIV Users
Izurieta 2019 [37]	Retrospective cohort	US	2017/18	Older adults (≥65 years)	Hospital encounters (inpatient hospitalizations/ER visits) for influenza (ICD-10: J09.xx, J10.xx, J11.xx and J129).	1,473,536	8,489,159
Izurieta 2020 [38]	Retrospective cohort	US	2018/19	Older adults (≥65 years)	Hospital encounters (inpatient hospitalizations/ER visits) for influenza (ICD-10: J09.xx, J10.xx, J11.xx and J129).	2,101,606	7,904,821
Izurieta 2021 [39]	Retrospective cohort	US	2019/20	Older adults (≥65 years)	Hospital encounters (inpatient hospitalizations/ER visits) for influenza (ICD-10: J09.xx, J10.xx, J11.xx and J129).	2,565,513	7,173,433
Machado 2021 [36]	Retrospective cohort	US	2016/17 2017/18	Older adults (≥65 years)	Hospitalizations/ER visits for influenza (ICD-9: 487–488 or ICD-10: J09–J11) and primary or secondary diagnosis of pneumonia (ICD-9: 480–486 or ICD-10: J12–J18)	4480 9061	182,786 113,895
Pelton 2020 [12]	Retrospective cohort	US	2017/18	Older adults (≥65 years)	Hospitalizations/ER visits for influenza (ICD-9: 487–488 or ICD-10: J09–J11)	234,313	1,269,855
Pelton 2021 [40]	Retrospective cohort	US	2018/19	Older adults (≥65 years)	Hospitalizations/ER visits for influenza (ICD-9: 487–488 or ICD-10: J09–J11)	561,243	1,672,797
Boikos 2021 [31]	Retrospective cohort	US	2017/18 2018/19	Older adults (≥65 years)	Medical encounters (outpatient visits, hospitalizations/ER visits) for influenza (ICD-9: 487–488 or ICD-10: J09–J11)	524,223 1,031,145	3,377,860 3,809,601
Levin 2021 [41]	Retrospective cohort	US	2019/20	Older adults (≥65 years)	Hospitalizations/ER visits for influenza (ICD-9: 487–488 or ICD-10: J09–J11)	798,255	1,654,162
Hsiao 2026 [42]	Cluster randomized crossover study	US	2023/24	Older adults (≥65 years)	PCR-confirmed influenza with hospitalization or ED access	212,875	216,720
McGovern 2024 [43]	Retrospective test-negative design	US	2017/18 2018/19 2019/20	Older adults (≥65 years)	LCI (hosp and/or ED) test-confirmed	619 1024 841	10,811 9400 6158
Imran 2025 [44]	Retrospective test-negative design	US	2022/2023	Older adults (≥65 years)	PCR-confirmed influenza with hospitalization or ED access	5853	11,162
Imran 2022 [45]	Retrospective cohort	US	2019/20	Adults ≥ 65 years	Influenza inpatient/ED; admitting diagnosis	936,508	1,813,819
Imran 2024 [46]	Retrospective cohort	US	2019/20	Adults ≥ 65 years; 0 or ≥1 influenza risk factor	Any influenza-related medical encounter; risk-stratum estimates only in this review	1,115,725	2,561,718
**Adjuvanted vaccines versus non-adjuvanted vaccines (standard dose)**							
**Authors**	**Study design**	**Country**	**Influenza season(s)**	**Study population**	**Primary influenza-related outcome(s)**	**N aTIV users**	**N standard TIV users**
Van Buynder 2013 [47]	Case control study design	Canada	2011/12	Older adults (≥65 years)	Testing positive for influenza	165	62
Pebody 2020 [48]	Case control study design	UK	2018/19	Older adults (≥65 years)	Laboratory-confirmed influenza hospitalization	639	282
Pott 2023 [35]	Test-negative design	Canada	2012/13 2013/14 2014/15	Older adults (≥65 years)	Laboratory-confirmed influenza-associated hospitalization among older adults, according to the vaccine type	156 184 186	1214 1232 918
Domnich 2022 [49]	Test-negative case-control study	Italy	2018/19 to 2019/20	Older adults (≥65 years)	Severe laboratory-confirmed influenza	141	282
Imran 2024 [50]	Retrospective cohort study	US	2019/2020	Adults ≥ 65 years with ≥1 influenza high-risk condition	Prevention of influenza-related medical encounters (IRMEs), outpatient IRMEs and influenza- and pneumonia-related hospitalizations	954,707	719,125
Ku 2024 [51]	Retrospective cohort	US	2022/23	Older adults (≥65 years)	Influenza-related medical encounters (positive influenza PCR test or influenza-related medical encounter in outpatient, inpatient, emergency or virtual visits identified by influenza ICD-10 codes without an influenza PCR-negative test ± 3 days of the code date) and PCR-confirmed influenza-related hospitalization (positive PCR test collected between −14 and +3 days from the inpatient admission date with an ARI code)	23,508	370,543
Boikos 2021 [31]	Retrospective cohort	US	2017/18 2018/19	Older adults (≥65 years)	Medical encounters (outpatient visits, hospitalizations/ER visits) for influenza (ICD-9: 487–488 or ICD-10: J09–J11)	524,223 1,031,145	917,609 (2017/18); 915,380 (2018/19)
Imran 2022 [45]	Retrospective cohort	US	2019/20	Adults ≥ 65 years	Influenza inpatient/ED; admitting diagnosis	936,508	651,034

## Data Availability

All data analyzed in this study were derived from previously published studies and are available in the cited articles and Supplementary Materials. The reconciled extraction, source-row provenance and reproducible R analysis scripts accompany this revision.

## Supplementary Materials

The following supporting information can be downloaded at https://www.mdpi.com/article/10.3390/vaccines14100883/s1, Table S1, PRISMA (preferred reporting items for systematic reviews and meta-analyses) checklist; Table S2, Automatic search strategy; Table S3A, ROBINS-I of studies estimating the relative vaccine effectiveness of adjuvanted influenza vaccines and nonadjuvanted high dose vaccines; Table S3B, ROBINS-I of estimating the relative vaccine effectiveness of adjuvanted influenza vaccines and standard dose vaccines; Table S4A, Risk of selective reporting of results estimating the relative vaccine effectiveness of adjuvanted influenza vaccines and nonadjuvanted high dose vaccines; Table S4B, Risk of bias in the included studies estimating the relative vaccine effectiveness of adjuvanted influenza vaccines and standard dose vaccines; Table S5A, Sensitivity to effect measure and estimate selection: ADJ vs. HD; Table S5B, Sensitivity to effect measure and estimate selection: ADJ vs. SD; Table S6A, Provenance of corrected overall estimates: ADJ vs. HD; Table S6B, Provenance of corrected overall estimates: ADJ vs. SD; Table S6C, Approved alternative overall estimates, retained without double counting; Table S7A, Verified subtype-specific estimates and observed seasons; Table S7B, Within-subtype sensitivity: Pott Table 2; Table S7C, Within-subtype sensitivity: Pott Table 3; Table S7D. Additional approved source estimates retained descriptively; Table S8A, Overall descriptive comparisons after data reconciliation; Table S8B, Native-measure restrictions; Table S8C, Three hospital test-negative studies; Table S8D, Two-publication high-dose age analyses; Table S8E, Three-publication broader-outcome high-dose age sensitivity; Table S9A, Subgroup meta-analysis of the relative vaccine effectiveness of MF59-adjuvanted trivalent influenza vaccine (aTIV) versus non-adjuvanted high-dose trivalent influenza vaccine (hdTIV) in adults aged ≥65 years, by age group; Table S9B, Standard-dose age analyses after comparator correction.

## Abbreviations

Preferred Reporting Items for Systematic Reviews and Meta-Analyses (PRISMA). MF59-adjuvanted vaccines (aTIV, aQIV). Non-adjuvanted high-dose vaccines (TIV-HD, hdQIV). Relative vaccine effectiveness (rVE). Vaccine effectiveness (VE).
