# Supplementary material for: Adjuvanted Influenza Vaccines Versus Other Influenza Vaccines in Adults Aged ≥60 Years: A Systematic Review and Meta-Analysis of Relative Effectiveness for Preventing Influenza-Related Hospitalization
Source: Vaccines (Basel). 2026 Oct 6;14(10):883. doi: 10.3390/vaccines14100883 (PMC13709410; doi:10.3390/vaccines14100883)
Supplement: Supplementary file 1 [file vaccines-14-00883-s001.zip › vaccines-4573304-supplementary.pdf]

Supplementary Table S1. PRISMA (preferred reporting items for systematic reviews and meta-analyses) checklist.

Section and Topic	Item #	Checklist item	Location where item is reported
**TITLE**			
Title	1	Identify the report as a systematic review.	Title
**ABSTRACT**			
Abstract	2	See the PRISMA 2020 for Abstracts checklist.	Abstract
**INTRODUCTION**			
Rationale	3	Describe the rationale for the review in the context of existing knowledge.	Section 1
Objectives	4	Provide an explicit statement of the objective(s) or question(s) the review addresses.	Section 1
**METHODS**			
Eligibility criteria	5	Specify the inclusion and exclusion criteria for the review and how studies were grouped for the syntheses.	Section 2.2
Information sources	6	Specify all databases, registers, websites, organisations, reference lists and other sources searched or consulted to identify studies. Specify the date when each source was last searched or consulted.	Section 2.3
Search strategy	7	Present the full search strategies for all databases, registers and websites, including any filters and limits used.	Section 2.3; Table S2
Selection process	8	Specify the methods used to decide whether a study met the inclusion criteria of the review, including how many reviewers screened each record and each report retrieved, whether they worked independently, and if applicable, details of automation tools used in the process.	Section 2.3
Data collection process	9	Specify the methods used to collect data from reports, including how many reviewers collected data from each report, whether they worked independently, any processes for obtaining or confirming data from study investigators, and if applicable, details of automation tools used in the process.	Section 2.3
Data items	10a	List and define all outcomes for which data were sought. Specify whether all results that were compatible with each outcome domain in each study were sought (e.g. for all measures, time points, analyses), and if not, the methods used to decide which results to collect.	Sections 2.2-2.3
	10b	List and define all other variables for which data were sought (e.g. participant and intervention characteristics, funding sources). Describe any assumptions made about any missing or unclear information.	Section 2.3

Section and Topic	Item #	Checklist item	Location where item is reported
Study risk of bias assessment	11	Specify the methods used to assess risk of bias in the included studies, including details of the tool(s) used, how many reviewers assessed each study and whether they worked independently, and if applicable, details of automation tools used in the process.	Section 2.4
Effect measures	12	Specify for each outcome the effect measure(s) (e.g. risk ratio, mean difference) used in the synthesis or presentation of results.	Section 2.6
Synthesis methods	13a	Describe the processes used to decide which studies were eligible for each synthesis (e.g. tabulating the study intervention characteristics and comparing against the planned groups for each synthesis (item #5)).	Sections 2.5-2.6
	13b	Describe any methods required to prepare the data for presentation or synthesis, such as handling of missing summary statistics, or data conversions.	Sections 2.5-2.6
	13c	Describe any methods used to tabulate or visually display results of individual studies and syntheses.	Sections 2.5-2.7
	13d	Describe any methods used to synthesize results and provide a rationale for the choice(s). If meta-analysis was performed, describe the model(s), method(s) to identify the presence and extent of statistical heterogeneity, and software package(s) used.	Sections 2.5-2.7
	13e	Describe any methods used to explore possible causes of heterogeneity among study results (e.g. subgroup analysis, meta-regression).	Sections 2.5-2.7
	13f	Describe any sensitivity analyses conducted to assess robustness of the synthesized results.	Sections 2.5-2.7
Reporting bias assessment	14	Describe any methods used to assess risk of bias due to missing results in a synthesis (arising from reporting biases).	NA
Certainty assessment	15	Describe any methods used to assess certainty (or confidence) in the body of evidence for an outcome.	NA
**RESULTS**			
Study selection	16a	Describe the results of the search and selection process, from the number of records identified in the search to the number of studies included in the review, ideally using a flow diagram.	Section 3.1; Figure 1
	16b	Cite studies that might appear to meet the inclusion criteria, but which were excluded, and explain why they were excluded.	Not reported individually
Study characteristics	17	Cite each included study and present its characteristics.	Section 3.1; Table 1

Section and Topic	Item #	Checklist item	Location where item is reported
Risk of bias in studies	18	Present assessments of risk of bias for each included study.	Section 3.2; Tables S3-S4
Results of individual studies	19	For all outcomes, present, for each study: (a) summary statistics for each group (where appropriate) and (b) an effect estimate and its precision (e.g. confidence/credible interval), ideally using structured tables or plots.	Sections 3.3-3.5; Tables S6-S7
Results of syntheses	20a	For each synthesis, briefly summarise the characteristics and risk of bias among contributing studies.	Sections 3.1-3.5; Tables S3-S4
	20b	Present results of all statistical syntheses conducted. If meta-analysis was done, present for each the summary estimate and its precision (e.g. confidence/credible interval) and measures of statistical heterogeneity. If comparing groups, describe the direction of the effect.	Sections 3.3-3.5; Tables S5-S9
	20c	Present results of all investigations of possible causes of heterogeneity among study results.	Sections 3.3-3.5; Tables S5-S9
	20d	Present results of all sensitivity analyses conducted to assess the robustness of the synthesized results.	Sections 3.3-3.5; Tables S5-S9
Reporting biases	21	Present assessments of risk of bias due to missing results (arising from reporting biases) for each synthesis assessed.	NA
Certainty of evidence	22	Present assessments of certainty (or confidence) in the body of evidence for each outcome assessed.	NA
**DISCUSSION**			
Discussion	23a	Provide a general interpretation of the results in the context of other evidence.	Discussion
	23b	Discuss any limitations of the evidence included in the review.	Strengths and Limitations
	23c	Discuss any limitations of the review processes used.	Strengths and Limitations
	23d	Discuss implications of the results for practice, policy, and future research.	Discussion; Conclusion
**OTHER INFORMATION**			

Section and Topic	Item #	Checklist item	Location where item is reported
Registration and protocol	24a	Provide registration information for the review, including register name and registration number, or state that the review was not registered.	Section 2.1
	24b	Indicate where the review protocol can be accessed, or state that a protocol was not prepared.	Section 2.1 (PROSPERO)
	24c	Describe and explain any amendments to information provided at registration or in the protocol.	Section 2.1
Support	25	Describe sources of financial or non-financial support for the review, and the role of the funders or sponsors in the review.	Funding statement
Competing interests	26	Declare any competing interests of review authors.	Conflicts of Interest
Availability of data, code and other materials	27	Report which of the following are publicly available and where they can be found: template data collection forms; data extracted from included studies; data used for all analyses; analytic code; any other materials used in the review.	Data Availability Statement

Supplementary Table S2. Automatic search strategy

#	Search term
	**MEDLINE, Biological Abstract and Global Health via OVID**
11	1. (fluad* OR MF59* OR "MF-59" OR "MF 59").mp.
2	2. exp Influenza Vaccines/ OR influenza vaccin*.mp.
3	OR ((influenza OR flu OR influenza virus*) adj5
4	(vaccin* OR immuni* OR inoculat*)).mp.
5	3. exp Influenza, Human/ OR influenza.mp.
6	4. exp Vaccines/ OR exp Viral Vaccines/ OR
7	Vaccines, Subunit/ OR Vaccines, Synthetic/ OR
8	vaccin*.mp. OR immuni*.mp.
9	5. 3 AND 4
10	6. exp Adjuvants, Immunologic/ OR adjuvant*.mp.
11	OR squalene*.mp. OR MF59*.mp.
12	OR polysorbate*.mp. OR emulsion*.mp.
13	7. (2 OR 5) AND 6
14	8. 1 OR 7
15	9. (high dose* OR high-dose* OR "high dose vaccin*" OR
16	Efluelda* OR Fluzone High-Dose* OR HD vaccin*).mp.
17	10. (standard dose* OR standard-dose* OR
18	"standard dose vaccin*" OR conventional dose* OR
19	conventional-dose* OR regular dose* OR
20	SD vaccin*).mp.
21	11. 8 AND (9 OR 10)
	**Web of Science**
1	#1 TS=(fluad* OR MF59* OR "MF-59" OR "MF 59")
2	#2 TS=("influenza vaccin*" OR influenza vaccin* OR ((influenza OR flu OR "influenza virus*") NEAR/5
3	(vaccin* OR immuni* OR inoculat*)))
4	#3 TS=(influenza OR "influenza, human")
5	#4 TS=(vaccin*OR immuni* OR "viral vaccin*"OR "subunit vaccin*" OR "synthetic vaccin*")
6	#5 #3 AND #4
7	#6 TS=(adjuvant* OR squalene*OR MF59* OR polysorbate*OR emulsion)

8	#7 (#2 OR #5) AND #6
9	#8 #1 OR #7
10	#9 TS=("high dose*" OR "high-dose*" OR "high dose vaccin*" OR Efluelda* OR "Fluzone High-Dose*" OR "HD vaccin*")
11	#10 TS=("standard dose*"OR "standard-dose*" OR "standard dose vaccin*" OR "conventional dose*"
12	#11 #8 AND (#9 OR #10)
	**Cochrane Library**
1	#1 (fluad* OR MF59* OR "MF-59" OR "MF 59"):ti,ab,kw
2	#2 ("influenza vaccin*" OR influenza vaccin* OR ((influenza OR flu OR "influenza virus*")
3	NEAR/5 (vaccin* OR immuni* OR inoculat*))):ti,ab,kw
4	#3 influenza:ti,ab,kw
5	#4 (vaccin* OR immuni* OR "viral vaccin*" OR"subunit vaccin*" OR "synthetic vaccin*"):ti,ab,kw
6	#5 #3 AND #4
7	#6 (adjuvant* OR squalene* OR MF59* OR polysorbate* OR emulsion*):ti,ab,kw
8	#7 (#2 OR #5) AND #6
9	#8 #1 OR #7
10	#9 ("high dose*" OR "high-dose*" OR "high dose vaccin*" OR Efluelda* OR "Fluzone High-Dose*" OR "HD vaccin*"):ti,ab,kw
11	#10 ("standard dose*" OR "standard-dose*" OR"standard dose vaccin*" OR "conventional dose*" OR

Supplementary Table S3. A ROBINS-I of studies estimating the relative vaccine effectiveness of adjuvanted influenza vaccines and nonadjuvanted high dose vaccines

	Item						
**Study**	**D1**	**D2**	**D3**	**D4**	**D5**	**D6**	**D7**
Izurieta et al., 2019	Moderate						Moderate
Izurieta et al., 2020	Moderate						Moderate
Izurieta et al., 2021	Moderate						Moderate
Machado et al., 2021	Moderate						Moderate
Pelton et al., 2020	Moderate						Moderate
Pelton et al., 2021	Moderate						Moderate
Boikos et al., 2021	Moderate						Moderate
Levin et al., 2021	Moderate						Moderate
Imran et al., 2025	Moderate						Moderate
Imran et al., 2022	Moderate						Moderate

McGovern et al., 2024	Moderate						Moderate
Hsiao et al., 2026	Moderate						Moderate
Imram et al., 2024	Moderate						Moderate

Supplementary Table S3. B ROBINS-I of estimating the relative vaccine effectiveness of adjuvanted influenza vaccines and standard dose vaccines.

	**Item**						
**Study**	**D1**	**D2**	**D3**	**D4**	**D5**	**D6**	**D7**
Van Buynder et al., 2013	Moderate						Moderate
Pebody et al., 2020	Moderate						Moderate
Pott et al., 2023	Moderate						Moderate
Domnich et al., 2022	Moderate						Moderate
Imran et al., 2024	Moderate						Moderate
Ku et al., 2024	Moderate						Moderate
Boikos 2021	Moderate						Moderate
Imram et al., 2024	Moderate						Moderate

D1 Bias due to confounding
D2 Bias in selection of participants into the study
D3 Bias in classification of interventions
D4 Bias due to deviations from intended interventions
D5 Bias due to missing data
D6 Bias in measurement of outcomes
D7 Bias in selection of the reported result

Supplementary Table S4. A Risk of selective reporting of results estimating the relative vaccine effectiveness of adjuvanted influenza vaccines and nonadjuvanted high dose vaccines

Study	Risk of confounding	Risk of selection bias	Risk of misclassification of exposure	Risk of deviation from intended vaccine	Risk of missing data	Risk of misclassification of outcomes	Risk of reporting bias	Overall risk of bias
Machado et al., 2021	Moderate	Low	Low	Low	Low	Low	Moderate	Moderate
Izurieta et al., 2019	Moderate	Low	Low	Low	Low	Moderate	Moderate	Moderate
Pelton et al., 2020	Moderate	Low	Low	Low	Low	Moderate	Moderate	Moderate
Boikos et al., 2021	Moderate	Low	Low	Low	Low	Moderate	Moderate	Moderate
Izurieta et al., 2020	Moderate	Low	Low	Low	Low	Low	Moderate	Moderate
Pelton et al., 2021	Moderate	Low	Low	Low	Low	Moderate	Moderate	Moderate
Izurieta et al., 2021	Moderate	Low	Low	Low	Low	Low	Moderate	Moderate
Levin et al., 2021	Moderate	Low	Low	Low	Low	Low	Moderate	Moderate
Imran et al., 2025	Moderate	Low	Low	Low	Low	Moderate	Moderate	Moderate
Imran et al., 2022	Moderate	Low	Low	Low	Low	Moderate	Moderate	Moderate
Imran et al., 2024	Moderate	Low	Low	Low	Low	Moderate	Moderate	Moderate
McGovern et al., 2024	Moderate	Low	Low	Low	Low	Low	Moderate	Moderate
Hsiao et al., 2026	Moderate	Low	Low	Low	Low	Low	Moderate	Moderate

Supplementary Table S4. B Risk of bias in the included studies estimating the relative vaccine effectiveness of adjuvanted influenza vaccines and standard dose vaccines

Study	Risk of confounding	Risk of selection bias	Risk of misclassification of exposure	Risk of deviation from intended vaccine	Risk of missing data	Risk of misclassification of outcomes	Risk of reporting bias	Overall risk of bias
Van Buynder et al., 2013	Moderate	Low	Low	Low	Low	Low	Moderate	Moderate
Pebody et al., 2020	Moderate	Low	Low	Low	Low	Moderate	Moderate	Moderate
Pott et al., 2023	Moderate	Low	Low	Low	Low	Moderate	Moderate	Moderate
Domnich et al., 2022	Moderate	Low	Low	Low	Low	Moderate	Moderate	Moderate
Imran et al., 2024	Moderate	Low	Low	Low	Low	Low	Moderate	Moderate

Ku et al., 2024	Moderate	Low	Low	Low	Low	Low	Moderate	Moderate
Boikos 2021	Moderate	Low	Low	Low	Low	Low	Moderate	Moderate
Imran et al., 2022	Moderate	Low	Low	Low	Low	Moderate	Moderate	Moderate

Supplementary Table S5A. Sensitivity to effect measure and estimate selection: ADJ vs HD.

One adjusted contribution per publication and scenario. Multi-publication rows use DerSimonian-Laird random effects and normal-theory intervals. Outcomes include explicitly labelled broader medical encounters; the existing hospital test-negative subset is reported separately. Mixed-measure summaries are exploratory and conditional on independence; they do not estimate a common causal risk ratio.

Analysis	Publications	Estimate [95% CI]	I^2
ADJ vs HD	12	0.977 [0.946; 1.010]	75.8%
ADJ vs HD only rate ratio	6	0.990 [0.953; 1.029]	84.1%
ADJ vs HD excluding rate ratio	6	0.939 [0.902; 0.977]	0.0%
ADJ vs HD only OR	4	0.940 [0.901; 0.981]	0.0%
ADJ vs HD excluding OR	8	0.986 [0.951; 1.023]	79.3%
ADJ vs HD only HR	2	0.931 [0.828; 1.048]	0.0%
ADJ vs HD excluding HR	10	0.980 [0.947; 1.014]	79.3%
HD alternative from workbook row 21	12	0.977 [0.945; 1.010]	75.8%
HD alternative from workbook row 22	12	0.976 [0.944; 1.009]	75.9%
HD alternative from workbook row 25	12	0.977 [0.945; 1.010]	75.8%
ADJ vs HD: rate ratios only, Machado Poisson model	7	0.991 [0.955; 1.029]	80.9%
HD Izurieta inpatient estimates replace composite outcomes	12	0.983 [0.946; 1.021]	76.6%
HD plus Imran 2024 no risk factors	13	0.976 [0.945; 1.007]	74.2%
HD plus Imran 2024 at least one risk factor	13	0.965 [0.924; 1.008]	89.7%

Supplementary Table S5B. Sensitivity to effect measure and estimate selection: ADJ vs SD.

Analysis	Publications	Estimate [95% CI]	I^2
ADJ vs SD	8	0.818 [0.783; 0.855]	41.6%
ADJ vs SD only OR	7	0.819 [0.792; 0.847]	26.6%
ADJ vs SD only HR	1	0.384 [0.180; 0.819]	Not pooled
SD without Imran hospital estimate	7	0.810 [0.772; 0.849]	41.9%
SD hospital test-negative ORs, Pott Table 2	3	0.693 [0.437; 1.099]	46.9%
SD hospital test-negative ORs, Pott Table 3	3	0.681 [0.503; 0.923]	17.5%

Analysis	Publications	Estimate [95% CI]	I²
SD alternative from workbook row 13	8	0.818 [0.782; 0.855]	41.6%
SD alternative from workbook row 14	8	0.816 [0.781; 0.853]	40.2%
SD alternative from workbook row 15	8	0.816 [0.781; 0.853]	40.2%
SD alternative from workbook row 16	8	0.779 [0.727; 0.834]	79.0%
SD alternative from workbook row 17	8	0.801 [0.749; 0.856]	68.2%
SD alternative from workbook row 19	8	0.816 [0.780; 0.853]	41.5%
SD alternative from workbook row 20	8	0.785 [0.721; 0.854]	76.2%
SD alternative from workbook row 21	8	0.776 [0.723; 0.834]	80.0%
SD alternative from workbook row 22	8	0.813 [0.778; 0.851]	43.2%

ADJ: adjuvanted; HD: high-dose; SD: standard-dose; OR: odds ratio; HR: hazard ratio. Rate ratios refer to source Poisson models. The single HR retains its source interval. Pott Table 3 adds frailty adjustment to the Table 2 comparison. Restricted sets also differ in design and population; they are not tests of interaction by measure. Selecting one estimate per publication does not establish independence across databases or seasons. Imran 2024 SD concerns adults with at least one high-risk condition. The two Imran 2024 HD risk strata enter separate sensitivity scenarios, not the overall-population reference display. Scenario inputs and the existing estimator/interval diagnostics are supplied in the R outputs.

Supplementary Table S6A. Provenance of corrected overall estimates: ADJ vs HD.

Study and seasons	Measure and design	Selected event	Source population / DOI
Izurieta 2019; 2017/18	rate ratio; retrospective cohort	Influenza hospitalization / ER; ADJ vs HD; Adults >=65	Medicare fee-for-service; 10.1093/infdis/jiy716
Izurieta 2020; 2018/19	rate ratio; retrospective cohort	Influenza hospitalization / ER; ADJ vs HD; Adults >=65	Medicare fee-for-service; 10.1093/infdis/jiaa080
Izurieta 2021; 2019/20	rate ratio; retrospective cohort	Influenza hospitalization / ER; ADJ vs HD; Adults >=65	Medicare fee-for-service; 10.1093/cid/ciaa1727
Levin 2021; 2019/20	rate ratio; retrospective cohort	Influenza hospitalization / ER; ADJ vs HD; Adults >=65	IQVIA linked claims (Dx, Rx, CDM); 10.3390/vaccines9101146
Pelton 2020; 2017/18	rate ratio; retrospective cohort	Influenza hospitalization / ER; ADJ vs HD; Adults >=65	IQVIA linked claims (Dx, Rx, CDM); 10.3390/vaccines8030446
Pelton 2021; 2018/19	rate ratio; retrospective cohort	Influenza hospitalization / ER; ADJ vs HD; Adults >=65	IQVIA linked claims (Dx, Rx, CDM); 10.1016/j.vaccine.2021.03.054
Imran 2022; 2019/20	OR; retrospective cohort	Influenza hospitalization / ER; ADJ vs HD; Adults >=65	Veradigm electronic records linked to claims; 10.1093/ofid/ofac167
Imran 2025; 2022/23	OR; test-negative study	Hospital encounters (inpatient + ER) test-confirmed influenza; ADJ vs HD; Adults >=65	Optum Market Clarity; 10.1016/j.ijid.2025.107983
McGovern 2024; 2017/18-2019/20	OR; test-negative study	LCI (hosp and/or ED) test-confirmed; ADJ vs HD; Adults >=65	US linked records; exact coverage not established from abstract; 10.1016/j.ijid.2024.107160
Hsiao 2026; 2023/24	HR; cluster randomized crossover trial	Hospitalized or ED PCR-confirmed; ADJ vs HD; Adults >=65	Kaiser Permanente Northern California; 10.1001/jamanetworkopen.2026.10120
Machado 2021; 2016/17-2017/18	HR; retrospective cohort	Influenza hospitalization / ER; ADJ vs HD; Adults >=65	MarketScan Medicare Supplemental; 10.1038/s41541-021-00373-w
Boikos 2021; 2017/18	OR; retrospective cohort	Any influenza-related medical encounter; ADJ vs HD; Adults >=65	Veradigm electronic records linked to claims; 10.1093/cid/ciab152

Comparisons are adjuvanted versus high-dose vaccine. Imran 2025 and McGovern 2024 were verified from published abstracts; full texts were unavailable. Original measures, DOI, source locations and numerical bounds are retained in the R data. Imran 2024 HD (10.1093/ofid/ofae459) is a distinct publication from Imran 2024 SD (10.1016/j.vaccine.2024.126316); its two approved risk-stratum estimates are listed in S6C and enter separate sensitivity scenarios.

Supplementary Table S6B. Provenance of corrected overall estimates: ADJ vs SD.

Study and seasons	Measure and design	Selected event	Source population / DOI
Domnich 2022; 2018/19 to 2019/20	OR; test-negative study	Laboratory-confirmed any-influenza hospitalization; ADJ vs SD; Adults >=65	IT-BIVE-HOSP hospital surveillance; 10.1016/j.ijid.2022.10.041
Imran 2022; 2019/20	OR; retrospective cohort	Influenza inpatient/ED encounter; admitting diagnosis; ADJ vs SD; Adults >=65	Veradigm electronic records linked to claims; 10.1093/ofid/ofac167
Pebody 2020; 2018/19	OR; test-negative study	Laboratory-confirmed influenza hospitalization; ADJ vs SD; Adults >=65	English hospital influenza surveillance; 10.1016/j.vaccine.2019.10.032
Pott 2023; 2012/13 to 2014/15	OR; test-negative study	Laboratory-confirmed influenza hospitalization; ADJ vs SD; Adults >=65	CIRN SOS hospital surveillance; 10.1016/j.vaccine.2023.08.070
Ku 2024; 2022/23	HR; retrospective cohort	Influenza hospitalization; ADJ vs SD; Adults >=65	Kaiser Permanente Southern California; 10.1093/cid/ciae375
Boikos 2021; 2017/18	OR; retrospective cohort	Any influenza-related medical encounter; ADJ vs SD; Adults >=65	Veradigm electronic records linked to claims; 10.1093/cid/ciab152
Van Buynder 2013; 2011/12	OR; test-negative study	laboratory confirmed influenza; ADJ vs SD; Adults >=65	Three Canadian health authorities; 10.1016/j.vaccine.2013.07.059
Imran 2024; 2019/2020	OR; retrospective cohort	influenza and pneumonia related hospitalizations; ADJ vs SD; Adults >=65 with at least one high-risk condition	Veradigm EHR linked to Komodo claims; 10.1016/j.vaccine.2024.126316

Comparisons are adjuvanted versus standard-dose vaccine. Pott contributes its direct comparative Table 2 OR, with Table 3 assessed separately; Domnich contributes its any-influenza estimate. Overlapping components are excluded from the overall comparisons, as described in Section 2.5. Source locations and numerical bounds are retained in the R data. Van Buynder contributes the community-based direct adjusted OR (Table 5). Imran 2024 SD contributes influenza/pneumonia hospitalization among adults with at least one high-risk condition; its broader IRME outcome is substituted in sensitivity analysis. Source numerical values are confirmed in the primary abstract (PMID 39299000); full text was not retrieved during this reconciliation. The immutable workbook and row-usage register retain original Excel worksheets and cells.

Supplementary Table S6C. Approved alternative overall estimates, retained without double counting.

Source row / study	Season / measure	Estimate [95% CI]	Outcome or alternative
Overall ADJ-SD!13; Pott 2023	2012/2013; OR	0.900 [0.630; 1.270]	Laboratory-confirmed influenza hospitalization; ADJ vs SD
Overall ADJ-SD!14; Pott 2023	2013/2014; OR	0.800 [0.560; 1.130]	Laboratory-confirmed influenza hospitalization; ADJ vs SD
Overall ADJ-SD!15; Pott 2023	2014/2015; OR	0.800 [0.580; 1.100]	Laboratory-confirmed influenza hospitalization; ADJ vs SD
Overall ADJ-SD!16; Imran 2022	2019/20; OR	0.725 [0.695; 0.756]	Influenza-related medical encounters; ADJ vs SD
Overall ADJ-SD!17; Imran 2024	2019/2020; OR	0.764 [0.739; 0.791]	influenza-related medical encounters (IRMEs); ADJ vs SD
Overall ADJ-SD!19; Boikos 2021	2017/2018; OR	0.812 [0.787; 0.837]	Any influenza-related medical encounter; ADJ vs SD; peak period
Overall ADJ-SD!20; Boikos 2021	2018/2019; OR	0.734 [0.706; 0.762]	Any influenza-related medical encounter; ADJ vs SD; peak period
Overall ADJ-SD!21; Imran 2022	2019/2020; OR	0.718 [0.687; 0.751]	Any influenza-related medical encounter; ADJ vs SD; peak period
Overall ADJ-SD!22; Pott 2023	2012/13 to 2014/15; OR	0.750 [0.610; 0.920]	Laboratory-confirmed influenza hospitalization; ADJ vs SD
Overall ADJ-HD!18; Izurieta 2019	2017/18; rate ratio	1.083 [1.054; 1.114]	Influenza hospitalization; ADJ vs HD
Overall ADJ-HD!19; Izurieta 2020	2018/19; rate ratio	0.986 [0.949; 1.025]	Influenza hospitalization; ADJ vs HD
Overall ADJ-HD!20; Izurieta 2021	2019/20; rate ratio	1.001 [0.961; 1.044]	Influenza hospitalization; ADJ vs HD
Overall ADJ-HD!21; Machado 2021	2016/17; HR	1.020 [0.570; 1.810]	Influenza hospitalization / ER; ADJ vs HD
Overall ADJ-HD!22; Machado 2021	2017/18; HR	0.940 [0.680; 1.310]	Influenza hospitalization / ER; ADJ vs HD
Overall ADJ-HD!23; Imran 2024	2019/20; OR	0.948 [0.849; 1.059]	Any influenza-related medical encounter; no risk factor; ADJ vs HD
Overall ADJ-HD!24; Imran 2024	2019/20; OR	0.875 [0.850; 0.900]	Any influenza-related medical encounter; at least 1 risk factor; ADJ vs HD
Overall ADJ-HD!25; Machado 2021	2016/17-2017/18; rate ratio	1.018 [0.766; 1.351]	Influenza hospitalization / ER; ADJ vs HD

Each alternative replaces the same publication in the corresponding scenario, except the two Imran 2024 HD strata, which are added separately to the reference display. No risk-stratum average is presented as an adjusted overall population OR. Workbook column L and the row-usage register provide the source cells and verified corrections.

Supplementary Table S7A. Verified subtype-specific estimates and observed seasons

Seven direct adjusted ORs from three hospital test-negative studies in adults aged ≥65 years. The outcome is hospitalization with laboratory-confirmed influenza of the specified subtype or type. Aggregate influenza A estimates are excluded.

Study / observed seasons	Subtype or type	Adjusted OR (95% CI)	Comparator and source
Domnich 2022; 2018/19 to 2019/20	A(H1N1)pdm09	0.453 (0.160 to 1.287)	aTIV versus egg-based QIV; Figure 1, p.168 (Domnich, 2022)
Pebody 2020; 2018/19	A(H1N1)pdm09	0.972 (0.206 to 4.581)	aTIV versus standard-dose IIV; Table 3, direct rVE (aTIV/IIV) (Pebody, 2020)
Pott 2023; 2012/13 to 2014/15	A(H1N1)pdm09	0.380 (0.160 to 0.760)	aTIV versus non-adjuvanted TIV; Table 2, p.6363 (Pott, 2023)
Domnich 2022; 2018/19 to 2019/20	A(H3N2)	0.431 (0.172 to 1.078)	aTIV versus egg-based QIV; Figure 1, p.168 (Domnich, 2022)
Pebody 2020; 2018/19	A(H3N2)	0.575 (0.142 to 2.337)	aTIV versus standard-dose IIV; Table 3, direct rVE (aTIV/IIV) (Pebody, 2020)
Pott 2023; 2012/13 to 2014/15	A(H3N2)	1.410 (1.110 to 1.760)	aTIV versus non-adjuvanted TIV; Table 2, p.6363 (Pott, 2023)
Pott 2023; 2012/13 to 2014/15	B (lineage unspecified)	0.940 (0.670 to 1.310)	aTIV versus non-adjuvanted TIV; Table 2, p.6363 (Pott, 2023)

Pott Table 2 is displayed; additional frailty adjustment is examined in S7C. Domnich uses the base-case propensity-matched and adjusted estimates, and Pebody the direct aTIV/IIV comparisons. OR = 1 - rVE/100, with reversed confidence limits, recovers the source OR without converting between effect measures. Domnich excluded 2020/21 and 2021/22 from effectiveness analyses. Pott does not identify the lineage of its B estimate.

Subtypes share test-negative controls within publications and their adjusted-estimate covariances were unavailable. No cross-subtype summary or test of subtype differences is reported (Cochrane Handbook, Chapter 6).

Supplementary Table S7B. Within-subtype sensitivity: Pott Table 2

Each subtype analysis contains one estimate from each of Pott, Domnich and Pebody (k = 3). The subtype analyses are not independent of each other.

Subtype (publications)	Estimator	OR	Normal 95% CI	HK 95% CI	Modified HK 95% CI	τ^2
A(H1N1)pdm09 (k = 3)	DL / REML / PM	0.457	0.256 to 0.816	0.176 to 1.186	0.128 to 1.630	0.00000
A(H3N2) (k = 3)	DL	0.794	0.324 to 1.944	0.153 to 4.134	0.111 to 5.669	0.43754
A(H3N2) (k = 3)	REML	0.809	0.350 to 1.868	0.154 to 4.233	0.129 to 5.081	0.36628
A(H3N2) (k = 3)	PM	0.834	0.392 to 1.777	0.159 to 4.388	0.159 to 4.388	0.27736

Supplementary Table S7C. Within-subtype sensitivity: Pott Table 3

Subtype (publications)	Estimator	OR	Normal 95% CI	HK 95% CI	Modified HK 95% CI	τ^2
A(H1N1)pdm09 (k = 3)	DL / REML / PM	0.492	0.273 to 0.889	0.209 to 1.159	0.134 to 1.803	0.00000
A(H3N2) (k = 3)	DL	0.761	0.378 to 1.530	0.201 to 2.875	0.164 to 3.525	0.22051
A(H3N2) (k = 3)	REML	0.762	0.381 to 1.525	0.202 to 2.881	0.167 to 3.490	0.21549
A(H3N2) (k = 3)	PM	0.799	0.440 to 1.453	0.215 to 2.969	0.215 to 2.969	0.13978

S7C changes only the Pott contribution to its additionally frailty-adjusted estimate. Calculations use log-transformed published ORs and SE = [log(upper CI) - log(lower CI)] / (2 × 1.96). Figure 9 retains the original source intervals, including asymmetry. Interval methods and grouped estimator labels follow Table S8; HK uses two degrees of freedom. All HK and modified HK intervals include 1. Three studies provide limited information about heterogeneity; these exploratory results do not establish equivalence or a consistent subtype-specific advantage (Röver, Knapp and Friede, 2015).

Supplementary TableS7D. Additional approved source estimates retained descriptively

Study / source row	Estimate type	Adjusted OR [95% CI]	Use
Pott 2023; row 8	B (lineage unspecified); Table 2	0.940 [0.670; 1.310]	Individual B estimate
Pott 2023; row 15	B (lineage unspecified); Table 3	0.780 [0.540; 1.110]	Individual B estimate
Pott 2023; row 16	Influenza A	0.840 [0.680; 1.030]	Aggregate A; excluded from subtype pooling
Domnich 2022; row 17	Influenza A	0.363 [0.171; 0.772]	Aggregate A; excluded from subtype pooling

Supplementary Table S8A. Overall descriptive comparisons after data reconciliation

The pre-existing DL, REML and Paule-Mandel comparisons were reproduced and updated on the corrected selection. These mixed-measure summaries contain one estimate per publication; participant overlap remains possible. The SD display contains eight publications and includes broader community and influenza/pneumonia outcomes; the HD reference display contains twelve overall-population estimates.

Comparison (publications)	Estimator	Ratio	Normal 95% CI	HK 95% CI	Modified HK 95% CI	τ^2
ADJ vs HD (k = 12)	DL	0.977	0.946 to 1.010	0.951 to 1.004	0.942 to 1.014	0.00195
ADJ vs HD (k = 12)	REML	0.979	0.951 to 1.007	0.952 to 1.006	0.948 to 1.011	0.00130
ADJ vs HD (k = 12)	PM	0.981	0.957 to 1.005	0.954 to 1.008	0.954 to 1.008	0.00079
ADJ vs SD (k = 8)	DL	0.818	0.783 to 0.855	0.766 to 0.875	0.766 to 0.875	0.00102
ADJ vs SD (k = 8)	REML	0.816	0.799 to 0.834	0.790 to 0.844	0.790 to 0.844	0.00000
ADJ vs SD (k = 8)	PM	0.746	0.611 to 0.910	0.586 to 0.948	0.586 to 0.948	0.04748

DL: DerSimonian-Laird; REML: restricted maximum likelihood; PM: Paule-Mandel; HK: Hartung-Knapp. Normal intervals use the standard normal quantile. HK uses a residual variance adjustment and t(k - 1); modified HK prevents the standard error from falling below its conventional value (Röver, Knapp and Friede, 2015; doi:10.1186/s12874-015-0091-1). Each estimator is shown separately; full-precision values are available in the R output. None of these methods corrects dependence or differences between effect measures. Prediction intervals in the R output use the corresponding normal or t(k - 1) quantile and the sum of between-study variance and variance of the estimated mean; small-k predictions remain unstable.

Supplementary Table S8B. Native-measure restrictions

Comparison (publications)	Estimator	Ratio	Normal 95% CI	HK 95% CI	Modified HK 95% CI	τ^2
ADJ vs HD only rate ratio (k = 6)	DL	0.990	0.953 to 1.029	0.954 to 1.028	0.942 to 1.041	0.00179
ADJ vs HD only rate ratio (k = 6)	REML	0.992	0.960 to 1.024	0.954 to 1.030	0.951 to 1.034	0.00113
ADJ vs HD only rate ratio (k = 6)	PM	0.992	0.964 to 1.022	0.955 to 1.032	0.955 to 1.032	0.00091
ADJ vs HD only OR (k = 4)	DL	0.940	0.901 to 0.981	0.885 to 0.998	0.877 to 1.007	0.00000
ADJ vs HD only OR (k = 4)	REML	0.940	0.901 to 0.981	0.885 to 0.998	0.877 to 1.007	0.00000
ADJ vs HD only OR (k = 4)	PM	0.940	0.901 to 0.981	0.885 to 0.998	0.877 to 1.007	0.00000
ADJ vs HD only HR (k = 2)	DL	0.931	0.828 to 1.048	0.512 to 1.695	0.433 to 2.005	0.00000
ADJ vs HD only HR (k = 2)	REML	0.931	0.828 to 1.048	0.512 to 1.695	0.433 to 2.005	0.00000
ADJ vs HD only HR (k = 2)	PM	0.931	0.828 to 1.048	0.512 to 1.695	0.433 to 2.005	0.00000
ADJ vs SD only OR (k = 7)	DL	0.819	0.792 to 0.847	0.781 to 0.859	0.781 to 0.859	0.00045
ADJ vs SD only OR (k = 7)	REML	0.817	0.800 to 0.834	0.792 to 0.842	0.792 to 0.842	0.00000
ADJ vs SD only OR (k = 7)	PM	0.809	0.720 to 0.910	0.699 to 0.937	0.699 to 0.937	0.01279

Selections follow Table S5. The single standard-dose HR is not pooled. Restricted sets differ in design and population. A rounded endpoint of 1.000 must not be used to determine significance.

Supplementary Table S8C. Three hospital test-negative studies

Comparison (publications)	Estimator	Ratio	Normal 95% CI	HK 95% CI	Modified HK 95% CI	τ^2
SD hospital test-negative ORs, Pott Table 2 (k = 3)	DL	0.693	0.437 to 1.099	0.270 to 1.776	0.252 to 1.906	0.08491
SD hospital test-negative ORs, Pott Table 2 (k = 3)	REML	0.690	0.431 to 1.105	0.268 to 1.776	0.246 to 1.938	0.09033
SD hospital test-negative ORs, Pott Table 2 (k = 3)	PM	0.707	0.465 to 1.074	0.282 to 1.772	0.282 to 1.772	0.06329
SD hospital test-negative ORs, Pott Table 3 (k = 3)	DL	0.681	0.503 to 0.923	0.355 to 1.308	0.350 to 1.328	0.02066

Comparison (publications)	Estimator	Ratio	Normal 95% CI	HK 95% CI	Modified HK 95% CI	τ^2
SD hospital test-negative ORs, Pott Table 3 (k = 3)	REML	0.666	0.469 to 0.947	0.329 to 1.351	0.308 to 1.441	0.03485
SD hospital test-negative ORs, Pott Table 3 (k = 3)	PM	0.687	0.516 to 0.915	0.367 to 1.288	0.367 to 1.288	0.01630

Both selections contain one direct comparative OR from Pott, Domnich and Pebody. Pott Table 3 adds frailty adjustment. All HK and modified HK intervals include 1. With three studies, heterogeneity and interval coverage are uncertain; these results do not establish equivalence.

Supplementary Table S8D. Two-publication high-dose age analyses

Comparison (publications)	Estimator	Ratio	Normal 95% CI	HK 95% CI	Modified HK 95% CI	τ^2
ADJ vs HD age 65-74 (k = 2)	DL	0.961	0.903 to 1.022	0.888 to 1.040	0.645 to 1.432	0.00000
ADJ vs HD age 65-74 (k = 2)	REML	0.961	0.903 to 1.022	0.888 to 1.040	0.645 to 1.432	0.00000
ADJ vs HD age 65-74 (k = 2)	PM	0.961	0.903 to 1.022	0.888 to 1.040	0.645 to 1.432	0.00000
ADJ vs HD age 75-84 (k = 2)	DL	0.968	0.905 to 1.036	0.641 to 1.463	0.626 to 1.498	0.00000
ADJ vs HD age 75-84 (k = 2)	REML	0.968	0.905 to 1.036	0.641 to 1.463	0.626 to 1.498	0.00000
ADJ vs HD age 75-84 (k = 2)	PM	0.968	0.905 to 1.036	0.641 to 1.463	0.626 to 1.498	0.00000
ADJ vs HD age >=85 (k = 2)	DL	1.027	0.932 to 1.132	0.546 to 1.931	0.546 to 1.931	0.00077
ADJ vs HD age >=85 (k = 2)	REML	1.027	0.932 to 1.132	0.546 to 1.931	0.546 to 1.931	0.00077
ADJ vs HD age >=85 (k = 2)	PM	1.027	0.932 to 1.132	0.546 to 1.931	0.546 to 1.931	0.00077

Pelton and Levin contribute one estimate each, using IQVIA data in successive seasons; absence of participant overlap has not been established. With k = 2, HK uses one degree of freedom. Zero estimated heterogeneity does not establish homogeneity, and these analyses do not test differences between age groups.

Supplementary Table S8E. Three-publication broader-outcome high-dose age sensitivity

Comparison (publications)	Estimator	Ratio	Normal 95% CI	HK 95% CI	Modified HK 95% CI	τ^2
HD broader outcome, age 65-74 (k = 3)	DL	0.943	0.908 to 0.980	0.900 to 0.988	0.867 to 1.025	0.00000
HD broader outcome, age 65-74 (k = 3)	REML	0.943	0.908 to 0.980	0.900 to 0.988	0.867 to 1.025	0.00000
HD broader outcome, age 65-74 (k = 3)	PM	0.943	0.908 to 0.980	0.900 to 0.988	0.867 to 1.025	0.00000
HD broader outcome, age 75-84 (k = 3)	DL	0.937	0.881 to 0.996	0.822 to 1.068	0.818 to 1.072	0.00120
HD broader outcome, age 75-84 (k = 3)	REML	0.937	0.880 to 0.997	0.822 to 1.068	0.817 to 1.074	0.00128
HD broader outcome, age 75-84 (k = 3)	PM	0.936	0.882 to 0.994	0.821 to 1.067	0.821 to 1.067	0.00104
HD broader outcome, age >=85 (k = 3)	DL	0.980	0.884 to 1.088	0.778 to 1.235	0.778 to 1.235	0.00477
HD broader outcome, age >=85 (k = 3)	REML	0.981	0.883 to 1.088	0.778 to 1.235	0.778 to 1.235	0.00480
HD broader outcome, age >=85 (k = 3)	PM	0.981	0.883 to 1.089	0.778 to 1.235	0.778 to 1.235	0.00494

The two Boikos seasons were first combined by fixed-effect inverse variance, then pooled with Pelton and Levin. This assumes independent seasonal estimates and combines broader medical encounters with hospital/ED outcomes. All modified HK intervals include 1. These small syntheses remain descriptive. Standard-

dose age analyses are excluded from this interval comparison because of repeated seasonal estimates; changing the estimator would not correct dependence. The wrong-comparator Pebody age rows have been removed; Boikos seasons remain separate as in the preceding analysis.

Supplementary Table S9A. Subgroup meta-analysis of the relative vaccine effectiveness of MF59-adjuvanted trivalent influenza vaccine (aTIV) versus non-adjuvanted high-dose trivalent influenza vaccine (hdTIV) in adults aged ≥65 years, by age group.

**Influenza-related endpoint**	65–74 years			75–84 years			≥85 years		
	Studies, n(estimates, n)	**rVE, % (95% CI)**	I^2, %	Studies, n(estimates, n)	**rVE, % (95% CI)**	I^2, %	Studies, n(estimates, n)	**rVE, % (95% CI)**	I^2, %
Influenza-related hospital encounters (inpatient stays and ER visits)	2 (2)	3.9 (−2.2 to 9.7)	0.0	2 (2)	3.2 (−3.6 to 9.5)	0.0	2 (2)	−2.7 (−13.2 to 6.8)	15.3

Primary subgroup pooling included Pelton et al. (2021) and Levin et al. (2021), which both evaluated influenza-related hospital or emergency department encounters. Each age-group model included two publications and two estimates. Boikos et al. (2021) was retained in broader-outcome sensitivity analyses because its outcome was any influenza-related medical encounter. Random-effects models used the DerSimonian-Laird estimator. Ratios below 1 and positive rVE values favour the adjuvanted vaccine. I^2, percentage of variability attributed to heterogeneity; rVE, relative vaccine effectiveness.

In an exploratory broader-outcome sensitivity analysis, the two Boikos seasons were first combined within study. Pooled ratios were 0.943 (95% CI 0.908 to 0.980; I^2 = 0%) for ages 65-74 years, 0.937 (95% CI 0.881 to 0.996; I^2 = 40.1%) for ages 75-84 years, and 0.980 (95% CI 0.884 to 1.088; I^2 = 56.4%) for ages 85 years or older. This sensitivity analysis combines broader medical encounters with hospital or emergency department outcomes.

Supplementary Table S9B. Standard-dose age analyses after comparator correction.

Age (years)	Publications (source estimates)	Adjusted OR [95% CI]	I^2
65-74	3 (4)	0.766 [0.694; 0.846]	89.5%
75-84	3 (4)	0.726 [0.667; 0.790]	76.9%
85 or older	3 (4)	0.761 [0.719; 0.806]	6.9%

Pott (2023), Boikos (2021) and Imran (2022) contribute. Pebody (2020) age estimates compared vaccination with no vaccination and are not direct ADJ/SD comparisons; they are excluded after source verification and confirmation by Daniela. The two Boikos seasons remain separate estimates, as in the preceding analysis. The only SD age data change is removal of Pebody. The DL normal-theory intervals remain conditional on treating the four rows as independent; within-publication correlation is not modelled. These OR summaries are exploratory.
